# Supplementary material for: Lanthanide-doped MoS2 with enhanced oxygen reduction activity and biperiodic chemical trends
Source: Nat Commun. 2023 Jun 5;14:3256. doi: 10.1038/s41467-023-39100-5 (PMC10241776; doi:10.1038/s41467-023-39100-5)
Supplement: Supplementary file 1 — Supplementary Information [file 41467_2023_39100_MOESM1_ESM.pdf]

Supplementary Information for

Lanthanide-Doped MoS_2 with Enhanced Oxygen Reduction Activity and Biperiodic Chemical Trends

Yu Hao,[1,2] Liping Wang,[1,2,*] and Liang-Feng Huang[1,2,3,*]

1. *Key Laboratory of Marine Materials and Related Technologies, Zhejiang Key Laboratory of Marine Materials and Protective Technologies, Ningbo Institute of Materials Technology and Engineering, Chinese Academy of Sciences, Ningbo 315201, China*
2. *Center of Materials Science and Optoelectronics Engineering, University of Chinese Academy of Sciences, Beijing 100049, China.*
3. *Research Center for Advanced Interdisciplinary Sciences, Ningbo Institute of Materials Technology and Engineering, Chinese Academy of Sciences, Ningbo 315201, China.*

*Corresponding Emails: *wangliping@nimte.ac.cn* ; *huangliangfeng@nimte.ac.cn*

Table of Contents

(A) Supplementary Structures and Stabilities of Ln-MoS_2 2

(B) Supplementary Raman Spectra of Ln-MoS_2 4

(C) Supplementary Electronic Properties of Ln Elements in Various States 6

(D) Supplementary Details of ORR Steps 11

(E) Supplementary Gibbs Free Energy of Reaction 12

(F) Supplementary Definition of Adsorption Free Energies 12

(G) Supplementary Data for the Effect of Water Environment 13

(H) Supplementary Energies and Electronic Structures for Adsorbed Ln-MoS_2 17

(I) Supplementary Details of the ORR Free Energy Diagrams for Ln-MoS_2 27

(J) Supplementary Formula and Results for Surface Pourbaix Diagrams 29

(K) Supplementary Details for the Microkinetic Model 33

(L) Supplementary Numerical Tests Validating the DFT Accuracy 44

(M) Supplementary References 48

(A) Supplementary Structures and Stabilities of Ln-MoS_2

The possible doping sites of Ln in MoS_2 include Mo, S, and interstitial sites. After systematic structural calculations, we find that the Ln substituent at a Mo site is the most stable, and the derived Ln-MoS_2 structure is stable against the structural relaxation. Apart from the stable Mo-site substitution, the other two dopant configurations are both unstable. The S-substituted Ln-MoS_2 cannot resist against any surface adsorption during the structural relaxation, where the Ln dopant can be automatically extracted out of the lattice by a surface adsorbate (**Figure S1**). During the structural relaxation of the interstitial-doped Ln-MoS_2, the interstitial Ln atom even automatically destroy the local crystal structure.

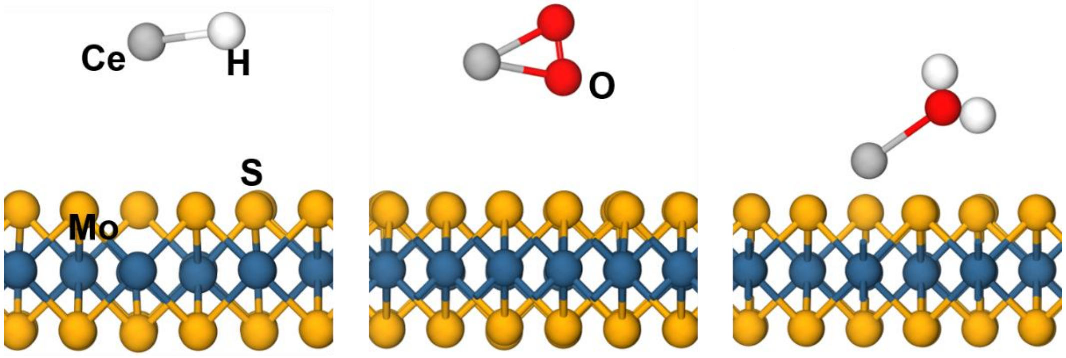

Figure S1. The adsorbed strucutres of Ln-MoS_2 with S atom substituted by the Ln dopant. The adsorbates include H, O_2, and H_2O.

The dynamical stability of the Mo-substituted Ln-MoS_2 systems is further proved by their phonon densities of states (**Figure S2**), where no imaginary phonon mode appears. The result on the favored stability of Mo-substituted Ln-MoS_2 is consistent with the experimental observation on synthesized Ln-MoS_2 samples by using scanning transmission electron microscopy (see the main text, Figure 1b). In the meantime, the Mo-substituted Ln-MoS_2 systems are also stable against the perturbation of different exterior adsorbates.

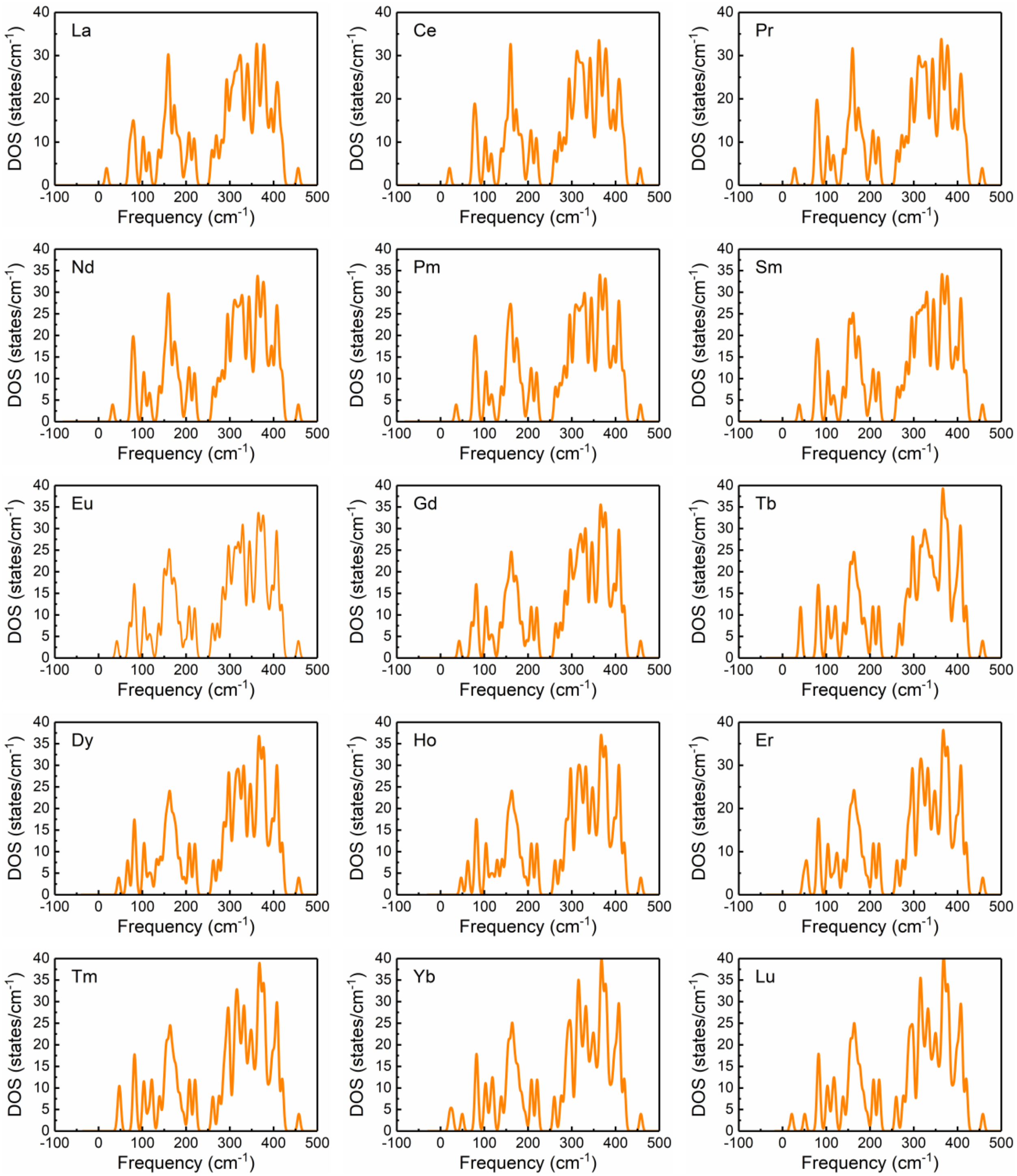

Figure S2. The phonon densities of states for all the fifteen Ln-MoS_2 structures. Source data are provided as a Source Data file.

(B) Supplementary Raman Spectra of Ln-MoS_2

The Raman spectra of pristine MoS_2 and Ln-MoS_2 (e.g., Er-MoS_2) have been measured in experiment [1]. It is necessary to study the effect of Ln dopant on the Raman spectra using accurate DFT calculation methods [2, 3], which can not only further prove the rationality of the used structural model here, but also guide more detailed experimental characterizations in the future. In the calculated Raman spectrum for the pristine MoS_2 (**Figure S3**), the positions of the two main Raman-active peaks, *i.e.*, the in-plane E_1^{2g} mode at 384.5 cm^{-1} and the out-of-plane A_{1g} mode at 406.7 cm^{-1}, are consistent with the experimental peaks at 384.2 ~ 385 and 403.8 ~ 404.9 cm^{-1} [4, 5], respectively.

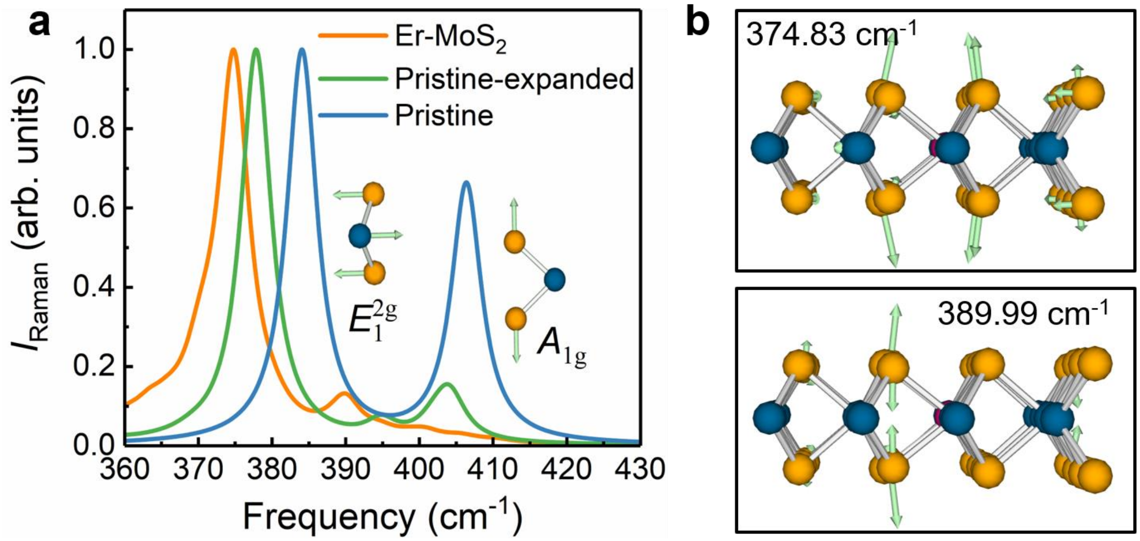

Figure S3. The calculated Raman spectra of Er-doped and pristine MoS_2 and the eigenvectors for the Raman-active modes. (a) The Raman spectra calculated from DFT for Er-MoS_2, pristine MoS_2, and the pristine MoS_2 with its lattice constant expanded to that of Er-MoS_2, where the two vibrational modes are shown alongside the two Raman peaks of pristine MoS_2 (blue curve). (b) The vibrational modes for the two main Raman peaks of Er-MoS_2 at 374.83 and 389.99 cm^{-1}, and there are obvious hybridizations between the in-plane and out-of-plane vibrations in each mode. Source data are provided as a Source Data file.

After Er doping, the two main Raman-active peaks in the calculated spectrum are both red shifted down to 374.8 and 390.0 cm^{-1} (**Figure S3**), which is consistent with the experimental reports [1]. To explore the reason for such Raman-peak redshift caused by Er doping, we further calculate the Raman spectrum of the pristine MoS_2 with the lattice constant expanded to be the same as that of Er-MoS_2. The results show that the lattice expansion caused by Er dopant only has a partial

contribution to the redshift, and the chemical effect of Er (e.g., changing the bond strength) should have an additional contribution. Furthermore, there are some vibraional hybridizations [6] occuring in the Raman-active modes, where each mode contains more or less both of the in-plane and out-of-plane vibrations. It is such vibrational hybridization that has made the higher Raman peak in pristine MoS_2 separated into many much lower peaks in both Er-MoS_2 and expanded MoS_2. The La and Lu dopants bring the largest and smallest lattice expansions to the MoS_2 lattice, respectively, and the Raman spectra of pristine MoS_2, Er-MoS_2, La-MoS_2, and Lu-MoS_2 are compared in **Figure S4**, where the qualitative correspondence between redshift of the lower peak and the excess area can be clearly seen.

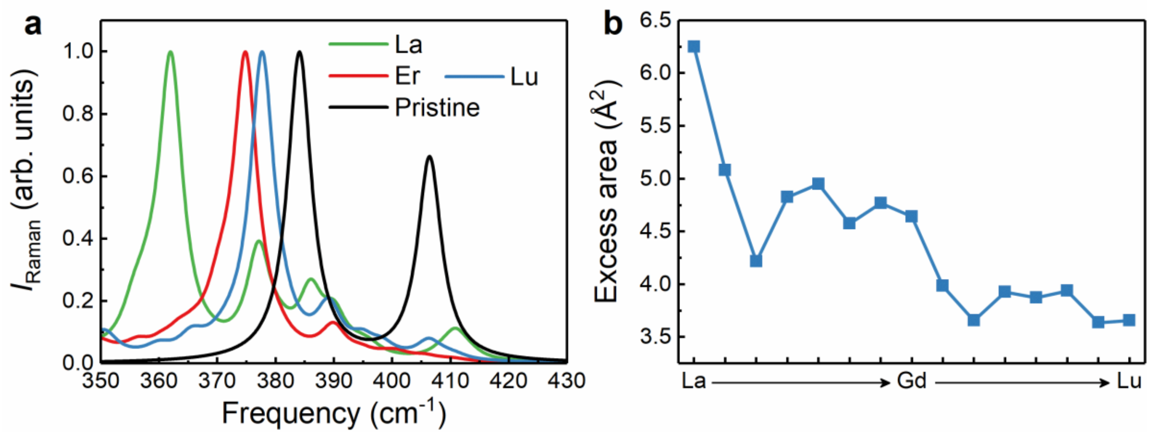

Figure S4. The calculated Raman spectra of Ln-MoS_2 and the excess areas caused by Ln doping in MoS_2. (a) The Raman spectra of La-, Er- and Lu-MoS_2 are choosen as the representatives to compare with that of pristine MoS_2. (b) The excess areas (i.e., expansion amounts in surface area) brought by Ln dopants in MoS_2. Source data are provided as a Source Data file.

(C) Supplementary Electronic Properties of Ln Elements in Various States

When in the state of atomic gas, the ground state valence electron configurations of La, Ce, Gd, and Lu atoms are $4f^{n-3}5d^{1}6s^{2}$ (n is the total number of 4f, 5d, and 6s electrons), and those of other Ln atoms are $4f^{n-2}6s^{2}$. The sublimation heats of elemental Ln metals [7] exhibit a biperiodic chemical trend with respect to Ln type (**Figure S5a**), which indicates the same trend for the interatomic bond strength in Ln metals. Similar biperiodic trend is also observed in the third and fourth ionization potentials of Ln atoms (*IP*, **Figure S5b**) [7], both of which can reflect the ionization capacity of 4f electrons. We make a weighted linear summation of $IP_3 + 0.2\times IP_4$, where the coefficients are first obtained by roughly fitting to the formation energies of Ln dopants, and then optimized to be 0.2 for a good trend presentation. The overall biperiodic trend of $IP_3 + 0.2\times IP_4$ is compared with the variation trend in the formation energy of Ln-MoS_2 (see main text, Figure 1c). Furthermore, the biperiodic trend also appears in the homolytic Ln–F bond energies of LnF_3 molecules (1/3LnF_3 → 1/3Ln + F, **Figure S6**) [8]. It is the common important role of 4f orbitals that have resulted in such coincident biperiodic trends in various physical properties of Ln in different states (e.g., atom, metal, and compound molecule).

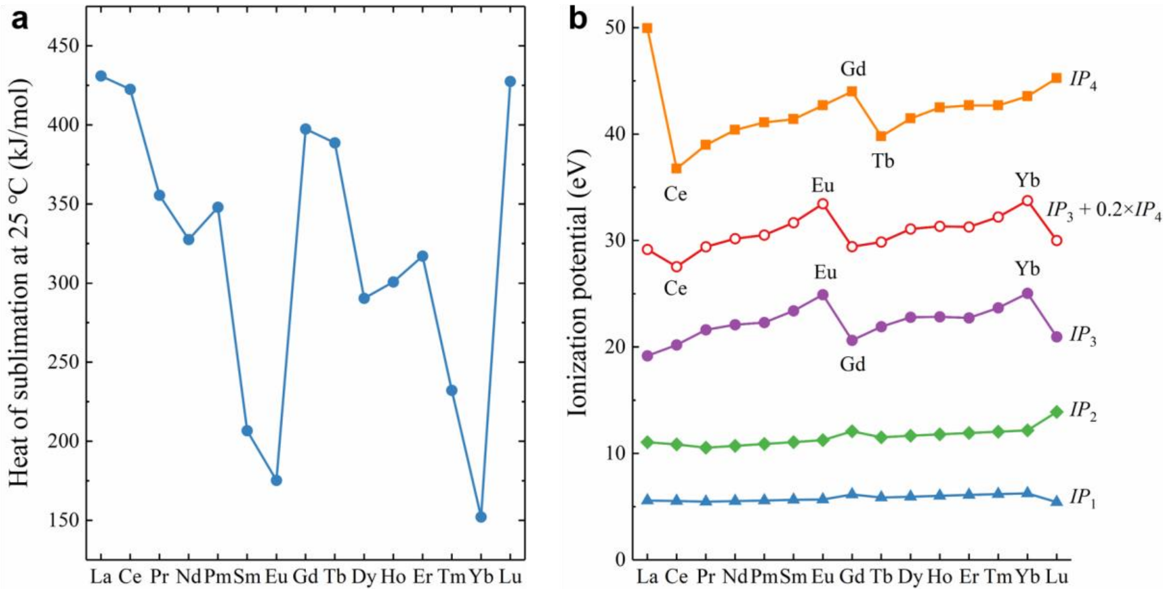

Figure S5. Sublimation heats of elemental Ln metals and the ionization potentials (*IP*) of Ln atoms. (a) Sublimation heats of elemental Ln metals at 25□. (b) The ionization potentials (*IP*) of Ln atoms [7]. We also make a weighted linear summation of *IP* ($IP_3 + 0.2\times IP_4$) to compare with the variation trend in the formation energy of Ln-MoS_2 (see main text, Figure 1c). Source data are provided as a Source Data file.

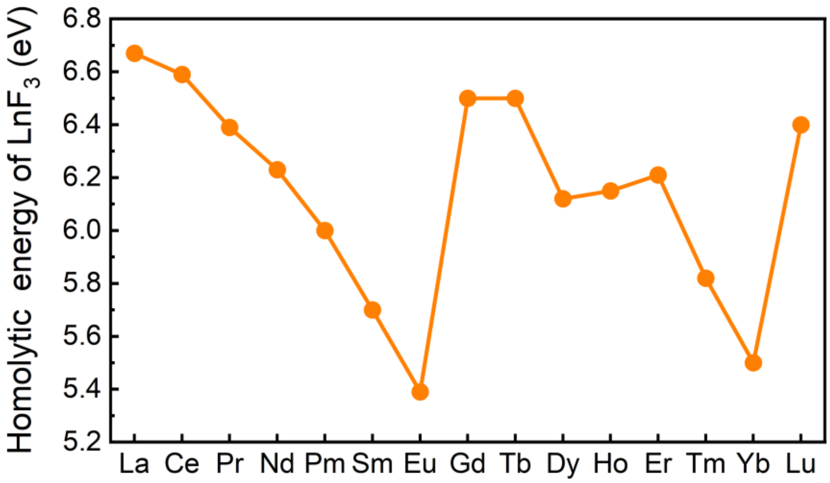

Figure S6. The homolytic Ln–F bond energies of LnF_3 molecules. The reaction equation is $1/3LnF_3 \rightarrow 1/3Ln + F$ [8]. The data for La, Ce, Pr, Nd, Eu, Gd, Dy, Ho, Er, and Tm are deduced from experimental measurements, and those for Pm, Sm, Tb, Yb, and Lu are obtained from quantum-chemistry calculations. Source data are provided as a Source Data file.

To clearly understand the intrinsic characters of atomic orbitals here, we perform the full-potential all-electron calculations for Ln atoms (residing at the center of a sphere with radius of 100 Å) using the pseudopotential generator (*i.e.*, the LD1 module) of the Quantum ESPRESSO code package [9]. The obtained energy levels and radial wavefunctions ($r\phi(r)$, r is the radial coordinate) of the 4*f*, 5*d*, and 6*s* orbitals are shown in **Figure S7a** and **S7b**. It can be seen that the 4*f* orbital of a Ln atom is clearly lower in energy and much localized in spatial distribution than those of the 5*d* and 6*s* orbitals. Thus, in a solid, it is the 5*d* and 6*s* orbitals on a Ln atom that can sufficiently bond with the orbitals of surrounding atoms. However, the highly-localized 4*f* orbitals have relatively weak bonding capability and mainly contribute to the magnetic moments of Ln-MoS_2 (**Figure S7c**), which also present a clear Hund-rule trend. The energy cost for the transition of 4*f* electrons up to the 5*d* and 6*s* orbitals [10] exhibit a biperiodic chemical trends with respect to the Ln type (**Figure S7d**). This can be well explained by an atomic-orbital mechanism:

(1) From La to Gd, the occupation of 4*f* orbitals approaches the half-filling state, the strong exchange attraction between electrons with the same spin orientation (*i.e.*, Hund rule) and the increased ion-electron attraction both contribute to the increased electronic-transition energy;

(2) When crossing the half-filling boundary from Eu to Gd, the electronic-transition energy decreased abruptly due to the additional 4*f* electron has no exchange attraction with other seven 4*f* electrons with different spin orientations;

(3) From Gd to Yb, the Hund rule is obeyed again by the added 4*f* electrons, and the ground-state is more and more stabilized by the increased electronic exchange attraction, as well as the increased ion-electron attraction.

The biperiodic chemical trend in the energy cost for the transition of 4*f* electrons up to 5*d* and 6*s* indicates that there should be a similar trend in the hybridization capability of 4*f* with 5*d* and 6*s*, which makes part of 4*f* electrons excited on to the later two orbitals. The exothermic interatomic bonding tend to increase with the degree of such endothermic intraatomic orbital hybridization [11]. It is such intrinsic orbital mechanism that has led to the general biperiodic trends ubiquitously appearing in the sublimation heats of Ln metals (**Figure S5a**), homolytic bond energies of LnF_3 molecules (**Figure S6**), and formation energies of Ln dopants in MoS_2 (see main text, Figure 1c).

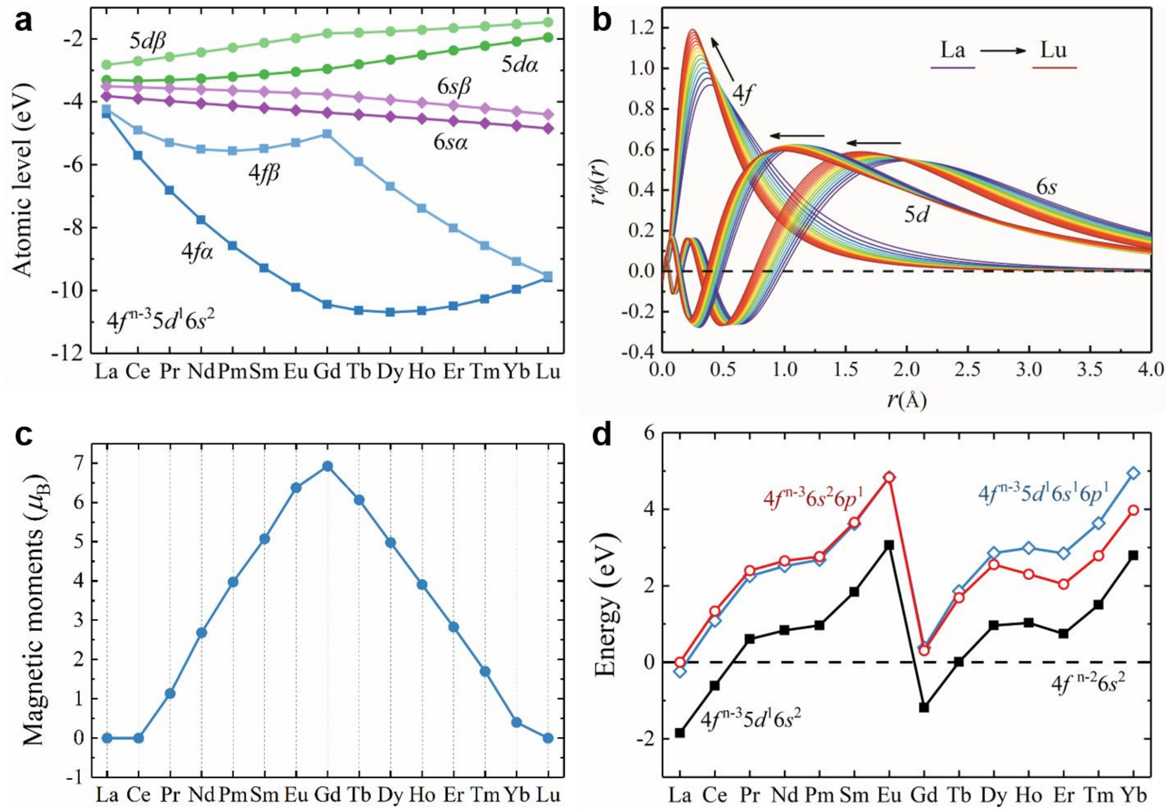

Figure S7. The intrinsic properties of Ln atom. (a, b) The energy levels and radial wave functions of the 5*d*, 6*s*, and 4*f* orbitals of isolated Ln atoms, where α and β denote different spin directions. (c) The total magnetic moments of Ln-MoS_2, which is mainly contributed by the Ln dopants. (d) The experimentally-measured relative energies between different orbital-occupation configurations of Ln atoms (reference is the $4f^{n-2}6s^2$ configuration) from Ref. [10]. Source data are provided as a Source Data file.

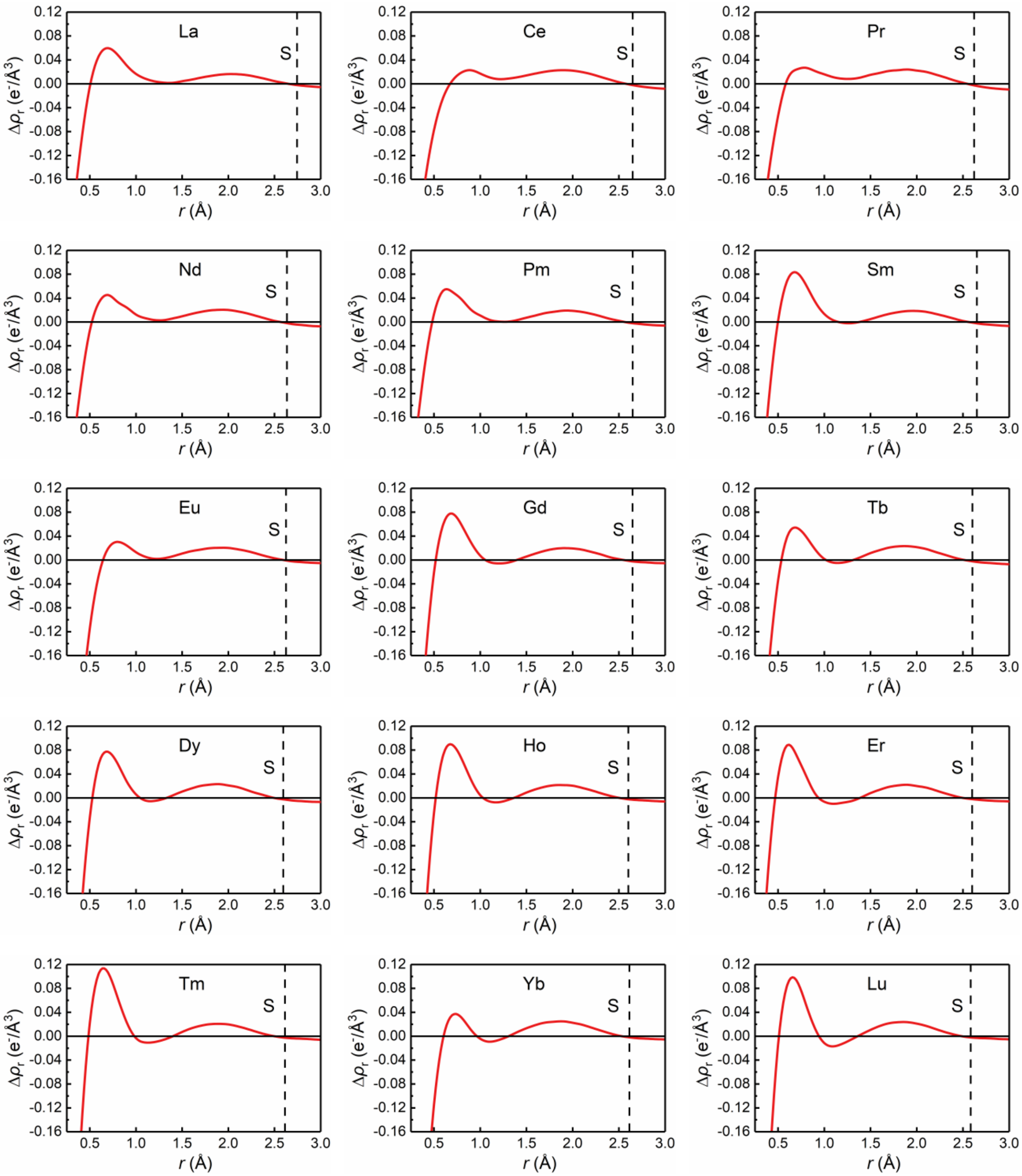

Figure S8. The radial distributions of the differential electronic densities ($\Delta\rho_r$) of all the fifteen Ln-MoS_2. The zero point (r = 0) is set at the dopant site. Source data are provided as a Source Data file.

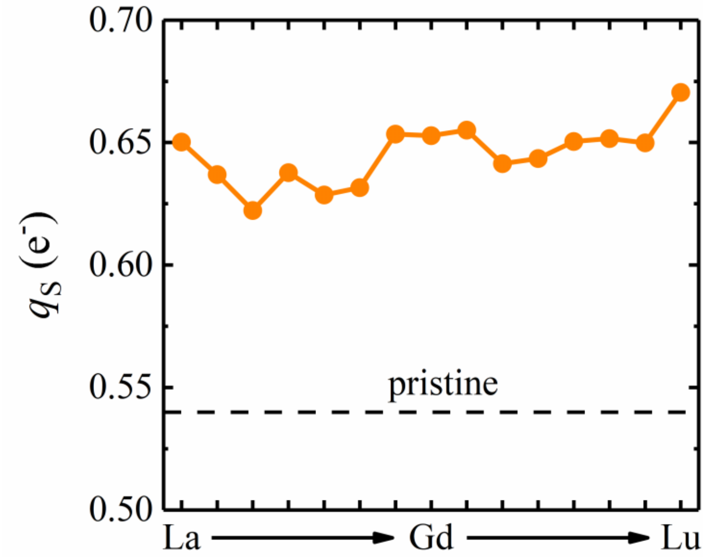

Figure S9. The q_S of the Ln-neighbor S atom in Ln-MoS_2. The dashed line is the q_S of a S atom in pristine MoS_2. Source data are provided as a Source Data file.

(D) Supplementary Details of ORR Steps

As the initial step of an ORR process, O_2 molecule first diffuses from the bulk electrolyte to the material-electrolyte interface, and then be adsorbed on an active S atom close to the Ln dopant. This process can be expressed as

(1) $O_2(aq) \underset{k_{-1}}{\overset{k_1}{\rightleftharpoons}} O_2(dl)$, and

(2) $O_2(dl) + *_A \underset{k_{-2}}{\overset{k_2}{\rightleftharpoons}} O_2{}^*{}_A$,

where $O_2(aq)$, $O_2(dl)$, and $O_2{}^*$ represent the O_2 molecule in aqueous solution, electrical double layer near the surface, and adsorbed state, respectively. k_i and k_{-i} are the rate constants for the forward and reverse reaction steps.

After being adsorbed on the Ln-MoS_2 surface, O_2 can be reduced into H_2O by electrochemical protonation steps, for which there are two possible mechanisms:

Mechanism 1: the associative path

(3) $O_2{}^*{}_A + H^+ + e^- \underset{k_{-3}}{\overset{k_3}{\rightleftharpoons}} OOH^*{}_A$,

(4) $OOH^*{}_A + H^+ + e^- \underset{k_{-4}}{\overset{k_4}{\rightleftharpoons}} O^*{}_A + H_2O$,

(5) $O^*{}_A + H^+ + e^- \underset{k_{-5}}{\overset{k_5}{\rightleftharpoons}} OH^*{}_A$,

(6) $OH^*{}_A + H^+ + e^- \underset{k_{-6}}{\overset{k_6}{\rightleftharpoons}} H_2O + *_A$,

Mechanism 2: the dissociative path

(7) $O_2{}^*{}_A + *_B \underset{k_{-7}}{\overset{k_7}{\rightleftharpoons}} O^*{}_A + O^*{}_B$,

(8) $O^*{}_B + H^+ + e^- \underset{k_{-8}}{\overset{k_8}{\rightleftharpoons}} OH^*{}_B$,

(9) $OH^*{}_B + H^+ + e^- \underset{k_{-9}}{\overset{k_9}{\rightleftharpoons}} H_2O + *_B$,

where $*_A$ and $*_B$ represent two adjacent surface S atoms closest to the Ln dopant. For OOH^*, in addition to being electrochemically reduced to H_2O in Eq. (4) above, it can also be chemically dissociated along the path of

(10) $OOH^*{}_A + *_B \underset{k_{-10}}{\overset{k_{10}}{\rightleftharpoons}} O^*{}_A + OH^*{}_B$.

(E) Supplementary Gibbs Free Energy of Reaction

The Gibbs free energy of reaction for each elemental ORR step (ΔG) is calculated using the computational hydrogen electrode (CHE) model [12]:

$$\Delta G = \Delta\varepsilon_0 + \Delta\varepsilon_{zpe} - T\Delta s + |e|U,$$

where $\Delta\varepsilon_0$ is the change in electronic energy; $\Delta\varepsilon_{zpe}$ is the change in vibrational zero-point energy; T is temperature (= 298.15 K here); and Δs is the entropy change. The last term is the contribution of electrode potential (U, referring to the reversible hydrogen electrode) and only needs to consider in the steps involving electron transfer. The entropies of clean/adsorbed surfaces are derived from the calculated vibrational frequencies, and those of gaseous molecules are collected from experimental data [13]. Liquid H_2O and gaseous H_2 are used as reference states, and the free energy of liquid H_2O is derived from that of the gaseous H_2O at 0.032 bar (i.e., the saturated-vapor-pressure of water at 298.15 K). The free energy of O_2 is obtained from the total reaction $O_2(g) + 2H_2(g) \rightarrow 2H_2O(l)$ with the reaction free energy of -4.92 eV at 1 bar and 298.15 K.

(F) Supplementary Definition of Adsorption Free Energies

The adsorption free energies (ΔG_{ads}) of O, OH, O_2, and OOH are calculated using the reaction equations of:

$H_2O + * \rightarrow O^* + H_2$,

$H_2O + * \rightarrow OH^* + 1/2H_2$,

$O_2 + * \rightarrow O_2^*$,

$2H_2O + * \rightarrow OOH^* + 3/2H_2$,

where the most stable adsorption sites are at the S atoms nearest to the Ln atom. The calculation method of reaction free energy here is consistent with that in Section (E).

(G) Supplementary Data for the Effect of Water Environment

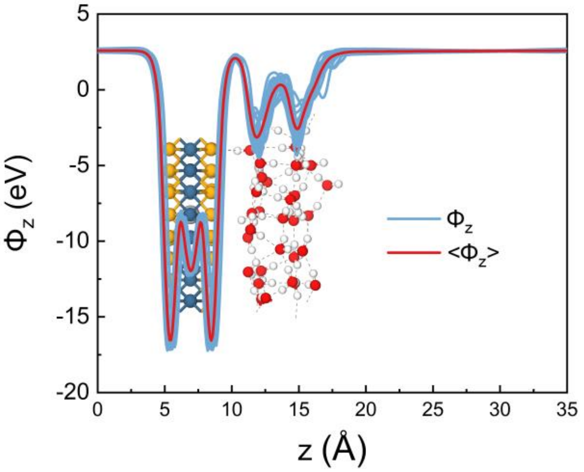

Figure S10. The one-dimensional electrostatic potential Φ_z of the water/Ce-MoS_2 slab. The direction is perpendicular to the water/Ce-MoS_2 interface. The red line shows the average of different water configurations ($<\Phi_z>$). For clarity, the geometry of the supercell along the surface normal is also included in the inset of the figure. Source data are provided as a Source Data file.

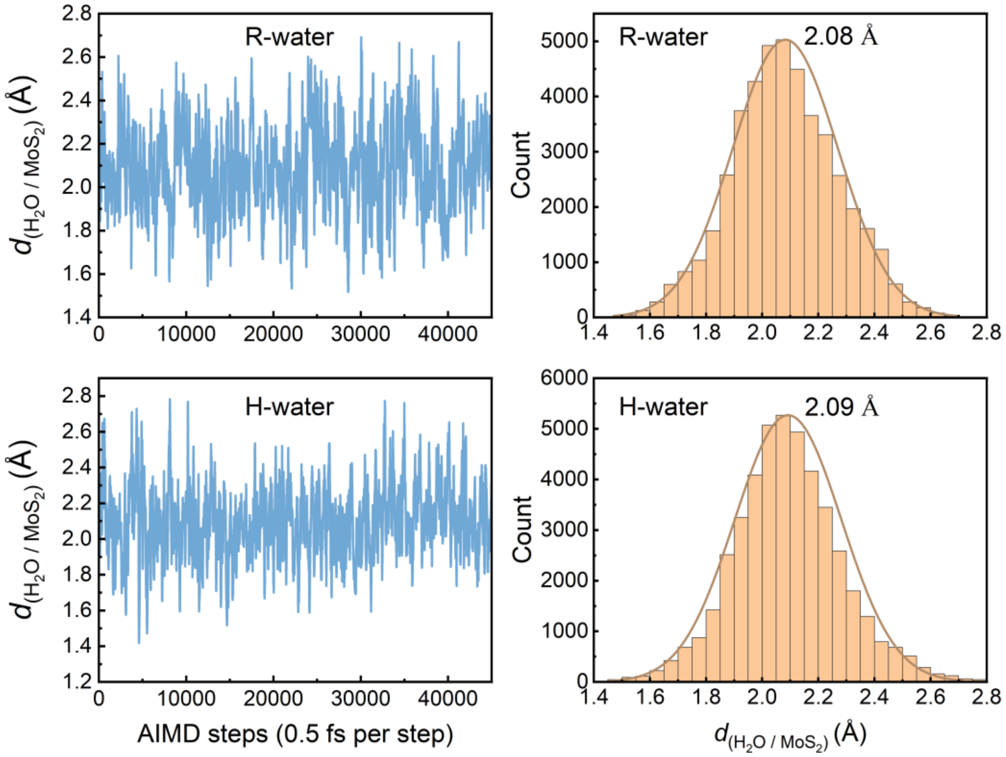

Figure S11. The interface distance between Ce-MoS_2 surface and water layer and the corresponding normal-distribution analyses. The distance is defined as the vertical-coordinate difference between the H atom in the H_2O molecule closest to the Ce-MoS_2 surface and the surface S atom. Source data are provided as a Source Data file.

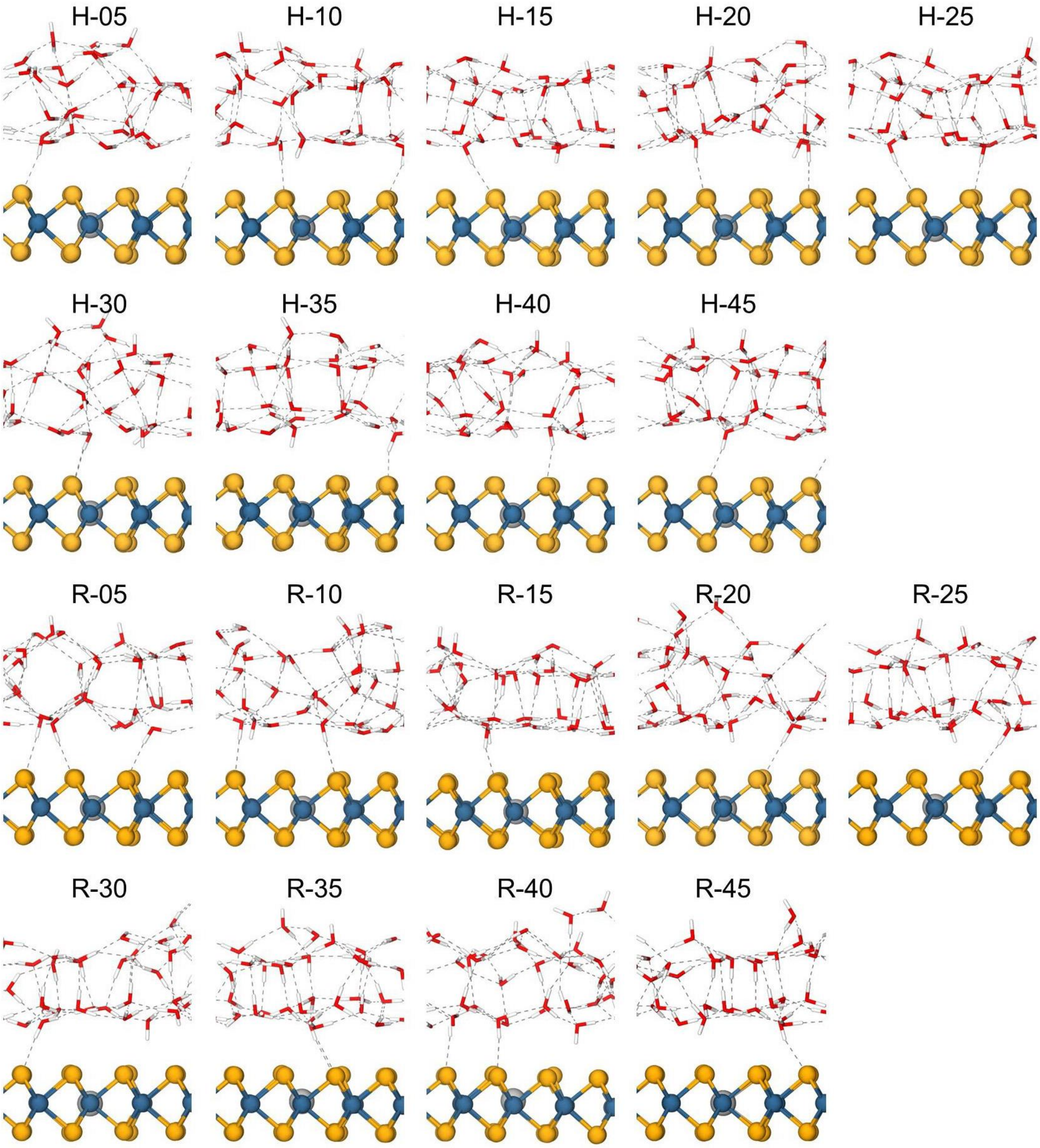

Figure S12. The sampled water structures calculated from the AIMD simulations. The samples are taken every 5000 steps and labeled as R-05 and H-05, etc.

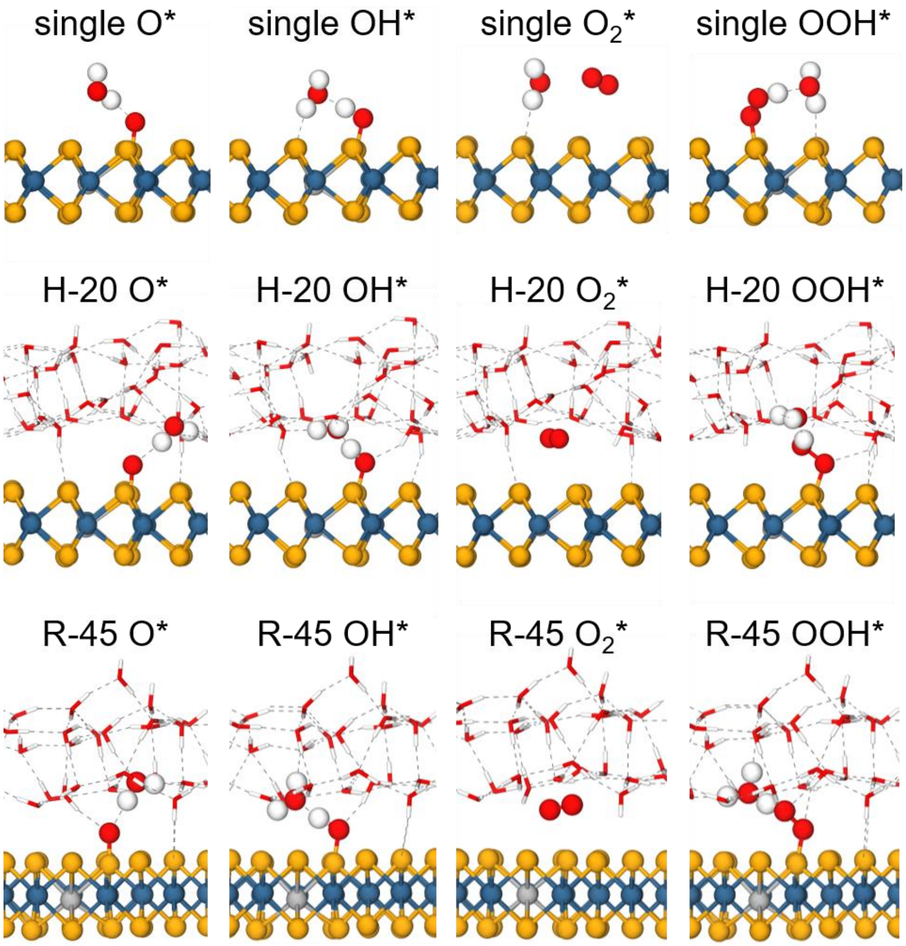

Figure S13. The adsorption structures of ORR intermediates on Ce-MoS$_2$. Single, H-20 and R-45 H_2O configurations are taken as representatives.

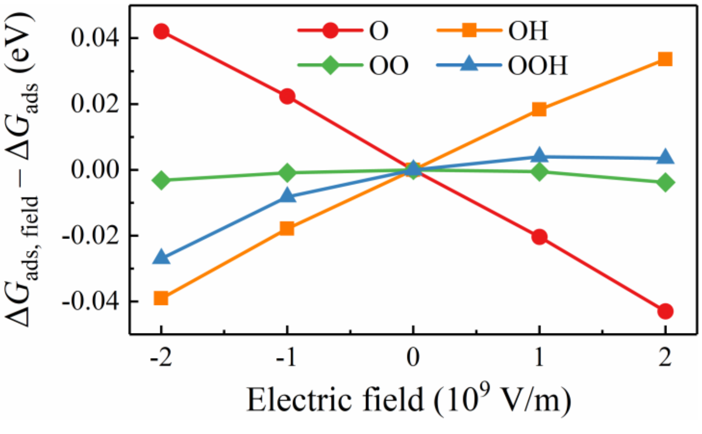

Figure S14. The difference of ΔG_{ads} with and without the applied electric field on Ce-MoS$_2$. Source data are provided as a Source Data file.

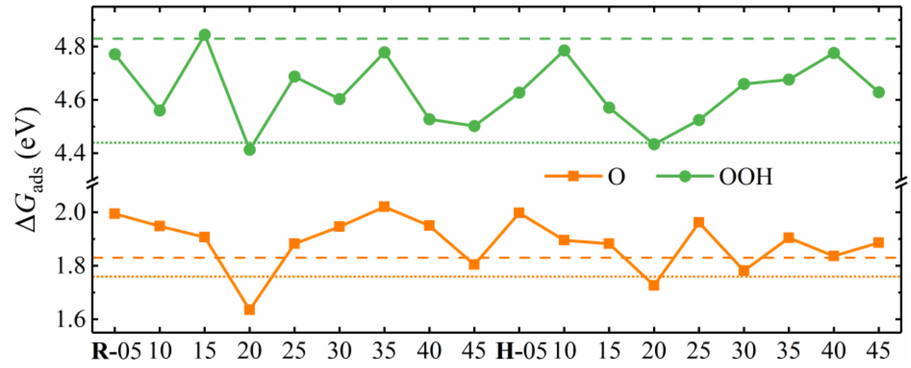

Figure S15. The ΔG_{ads}'s of O and OOH with different water configurations on Ce-MoS_2. The dashed and dotted lines represent the ΔG_{ads}'s for the environments of vacuum and a single H_2O, respectively. Source data are provided as a Source Data file.

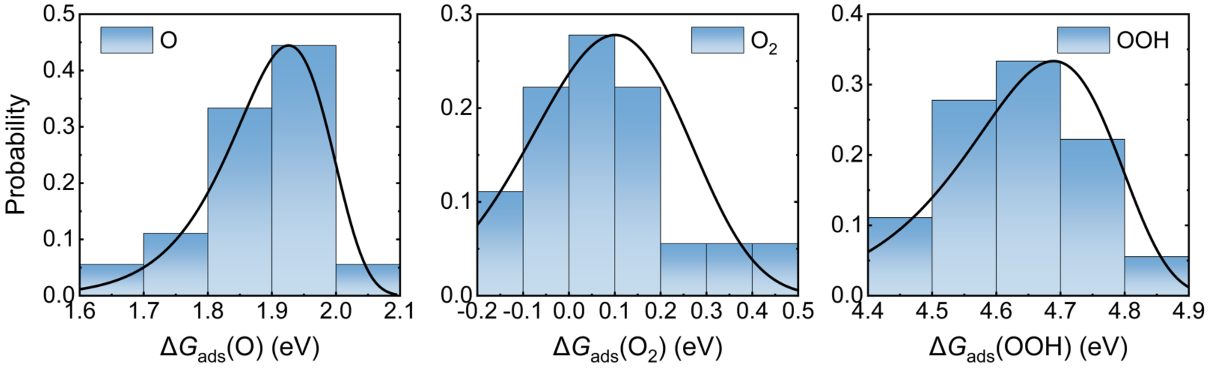

Figure S16. The distributions of ΔG_{ads}'s with different water configurations. They exhibit the characteristics of left skewed Weibull distribution. Source data are provided as a Source Data file.

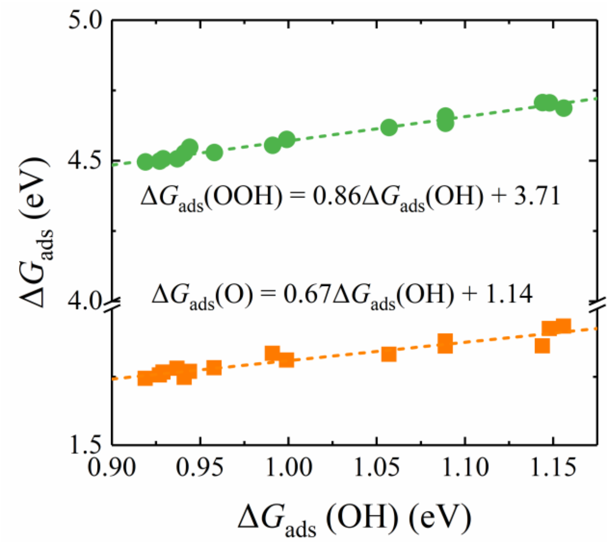

Figure S17. The linear relationships of ΔG_{ads}(O) and ΔG_{ads}(OOH) with ΔG_{ads}(OH) in the water environment. Source data are provided as a Source Data file.

(H) Supplementary Energies and Electronic Structures for Adsorbed Ln-MoS_2

The ΔG_{ads}s for different adsorbates on Ln-MoS_2 surfaces, except for $\Delta G_{ads}(O_2)$, clearly exhibit the biperiodic trends (**Figure S18**). When comparing the doping-induced ΔG_{ads} changes ($\Delta\Delta G_{ads}$, with respect to the ΔG_{ads} on pristine MoS_2) for different adsorbates on all the fifteen Ln-MoS_2 surfaces, we can find that all the fifteen Ln dopants have a quite uniform chemical tuning effect (**Figure S19**, left). For example, after any kind of Ln doping, $\Delta G_{ads}(OH)$ is decreased by a large amount of magnitude (1.18 ~ 1.49 eV), $\Delta G_{ads}(OOH)$ is moderately decreased (by 0.22 ~ 0.53 eV), and both $\Delta G_{ads}(O)$ and $\Delta G_{ads}(O_2)$ are always marginally decreased (by 0.02 ~ 0.26 and 0.03 ~ 0.14 eV, respectively). Similarly, the water effect on ΔG_{ads} also has a uniform energetic effect for the adsorbates on all the fifteen Ln-MoS_2 surfaces (**Figure S19**, right). For example, the water-induced decreases in $\Delta G_{ads}(OH)$ (by 0.19 ~ 0.35 eV) and $\Delta G_{ads}(OOH)$ (by 0.01 ~ 0.17 eV) and increases in $\Delta G_{ads}(O)$ (by 0.03 ~ 0.18 eV) and $\Delta G_{ads}(O_2)$ (by 0.15 ~ 0.29 eV) are distinctively separated. Therefore, these uniform effects of Ln doping and water environment can be succinctly reflected by the changes in the average ΔG_{ads}s over all the fifteen Ln-MoS_2 systems, as shown in Figure 2g.

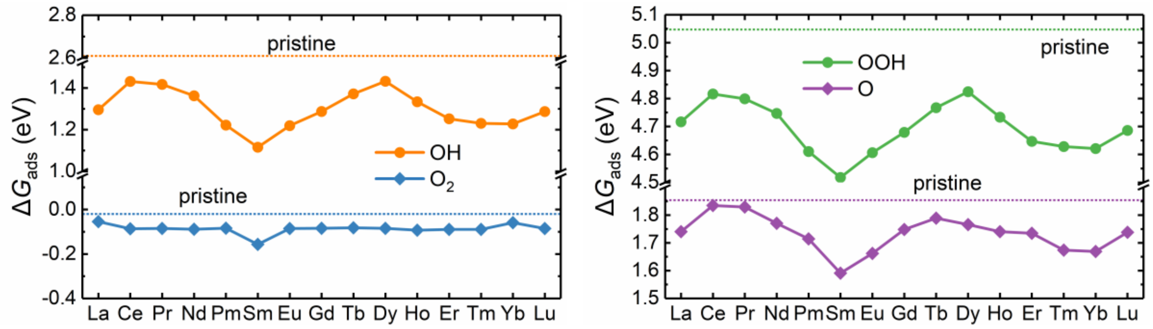

Figure S18. The ΔG_{ads}s of different adsorbates on all the fifteen Ln-MoS_2 surfaces in vacuum. Source data are provided as a Source Data file.

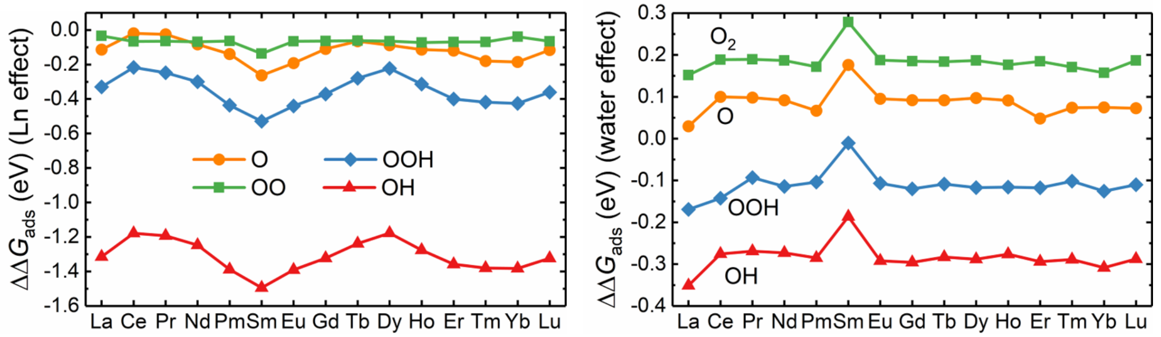

Figure S19. The effects of Ln doping and water environment on the ΔG_{ads}s for different adsorbates on all the fifteen Ln-MoS_2 systems. Source data are provided as a Source Data file.

The adsorption structures and the differential electronic densities induced by the adsorption of ORR intermediates on the pristine MoS_2 and Ln-MoS_2 surfaces are shown in **Figure S20**. It can be seen that the length of S−O bond in the OH^* system is considerably shortened from 1.81 down to 1.65 Å after Ln doping, while that in the O^* system has been barely changed. In addition, the adsorption state of OOH has a special change from physical adsorption to chemical adsorption after Ln doping. These changes in bond lengths are in accordance with the selectively enhanced adsorption of OH and OOH by Ln doping, as discussed in the main text. From the differential electronic densities on Ln-MoS_2, we can clearly observe the highly hybridized atomic orbitals around the S−O bond in O^*, OH^*, and OOH^* on Ln-MoS_2, with a higher hybridization degree than that on pristine MoS_2, indicating the stronger covalent S−O bonding on Ln-MoS_2.

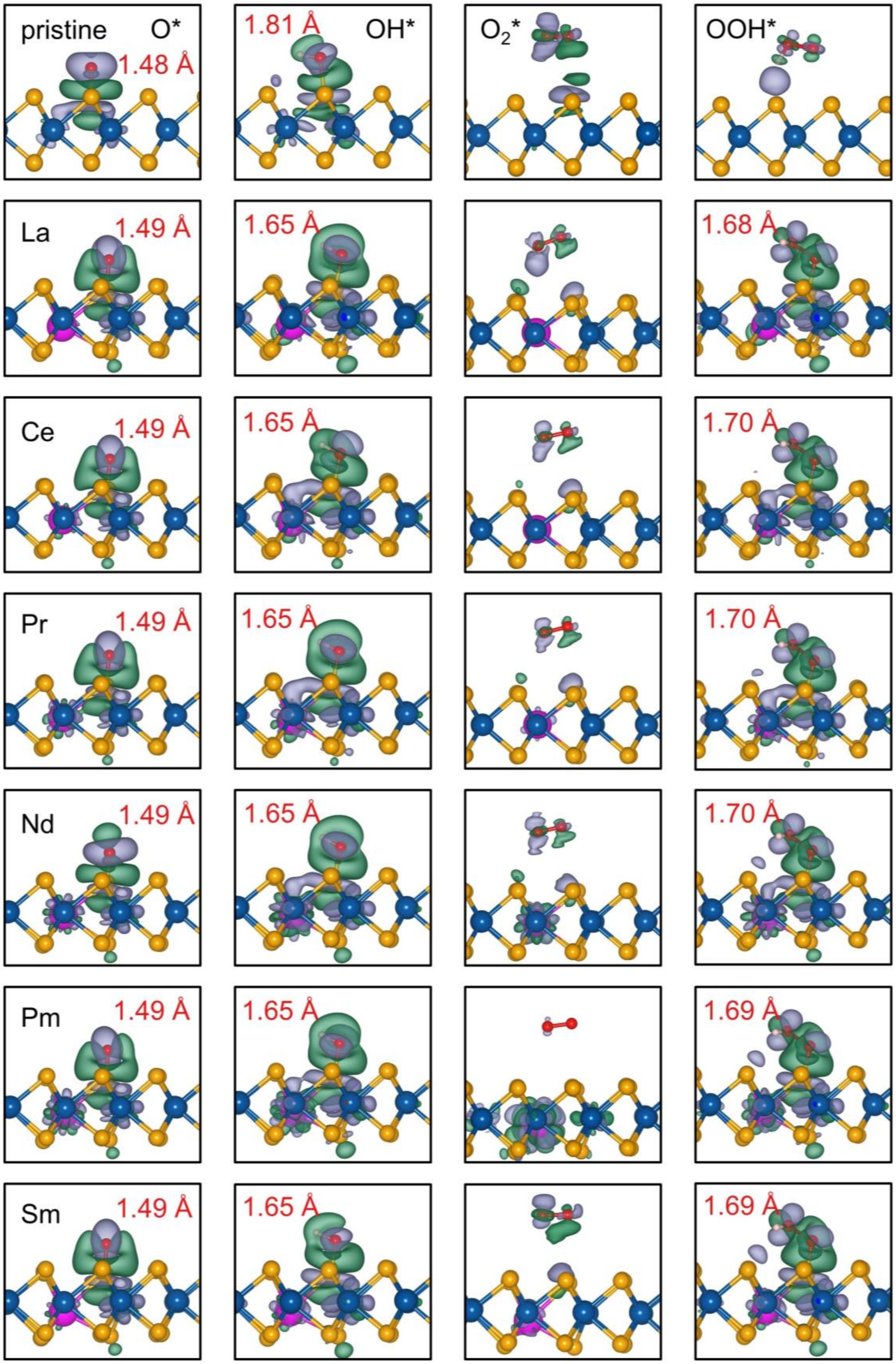

pristine
O*
1.48 Å
1.81 Å
OH*
O_2*
OOH*
La
1.49 Å
1.65 Å
1.68 Å
Ce
1.49 Å
1.65 Å
1.70 Å
Pr
1.49 Å
1.65 Å
1.70 Å
Nd
1.49 Å
1.65 Å
1.70 Å
Pm
1.49 Å
1.65 Å
1.69 Å
Sm
1.49 Å
1.65 Å
1.69 Å

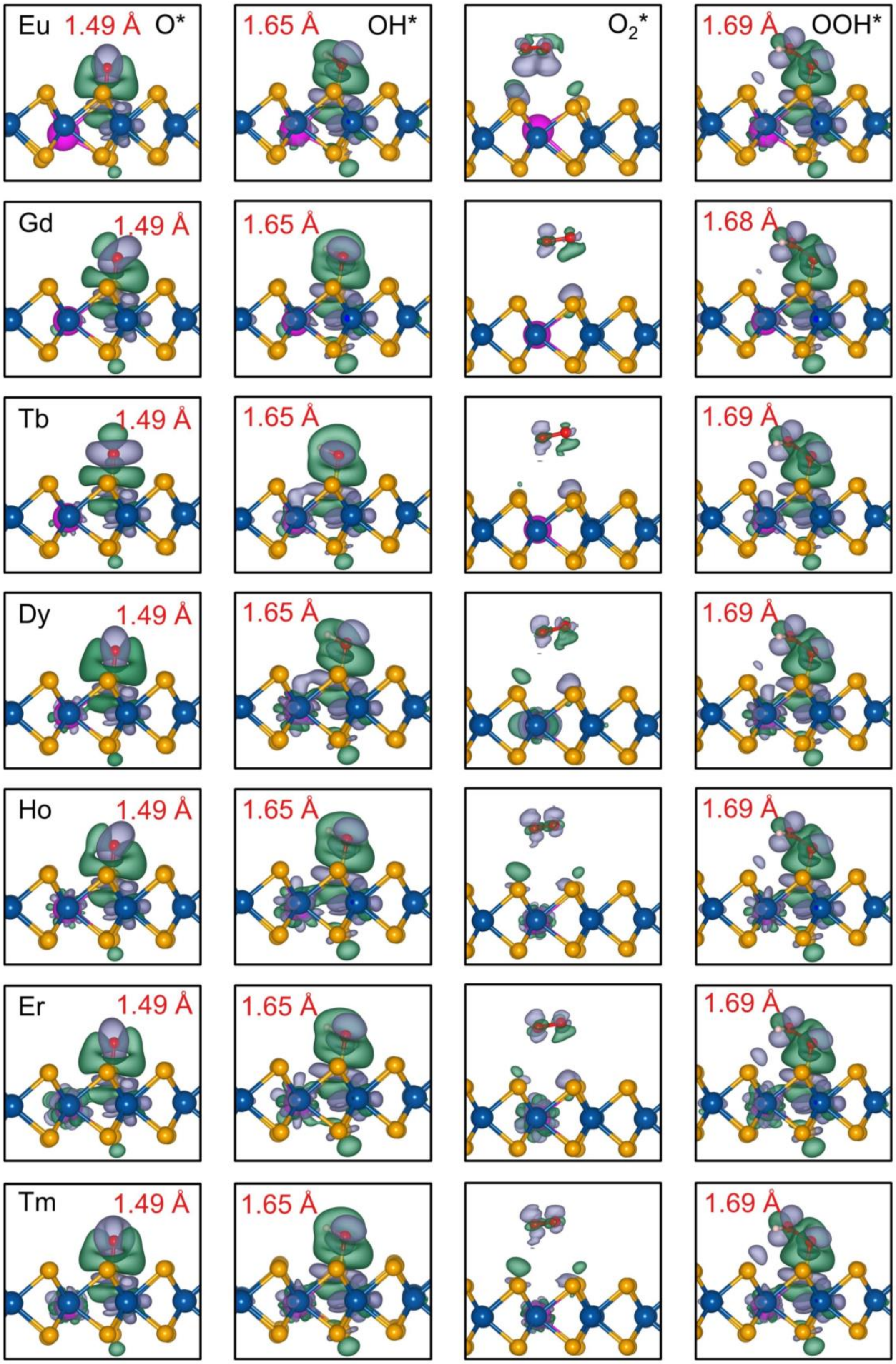
Eu
1.49 Å
O*
1.65 Å
OH*
O$_2$*
1.69 Å
OOH*
Gd
1.49 Å
1.65 Å
1.68 Å
Tb
1.49 Å
1.65 Å
1.69 Å
Dy
1.49 Å
1.65 Å
1.69 Å
Ho
1.49 Å
1.65 Å
1.69 Å
Er
1.49 Å
1.65 Å
1.69 Å
Tm
1.49 Å
1.65 Å
1.69 Å

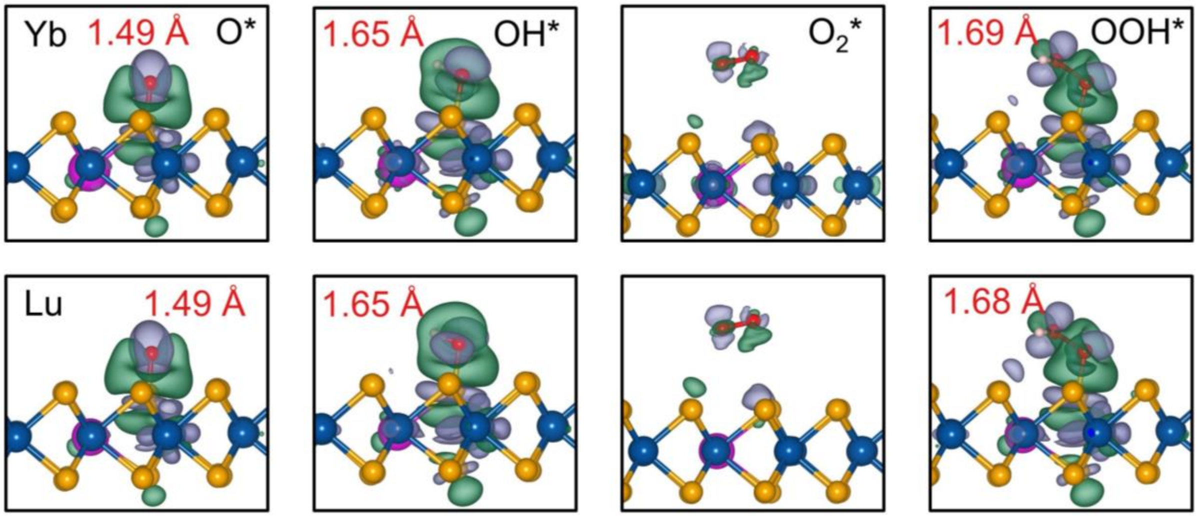

Figure S20. The adsorption structures of O, OH, O_2, and OOH on pristine MoS_2 and Ln-MoS_2 and the differential electronic densities induced by these adsorbates. The purple and green regions represent the accumulation and depletion of electronic density, respectively. The length of S–O bonds in O^*, OH^*, and OOH^* systems are also labeled.

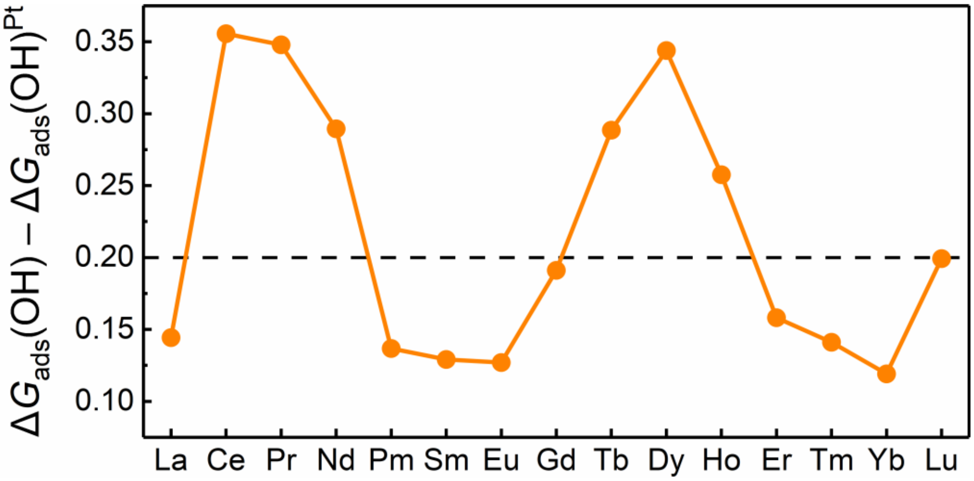

Figure S21. The relative values of ΔG_{ads}(OH)s for Ln-MoS_2 surfaces with respect to that of Pt (111) surface. The ΔG_{ads}(OH) of Pt (111) is 0.80 eV [12]. Source data are provided as a Source Data file.

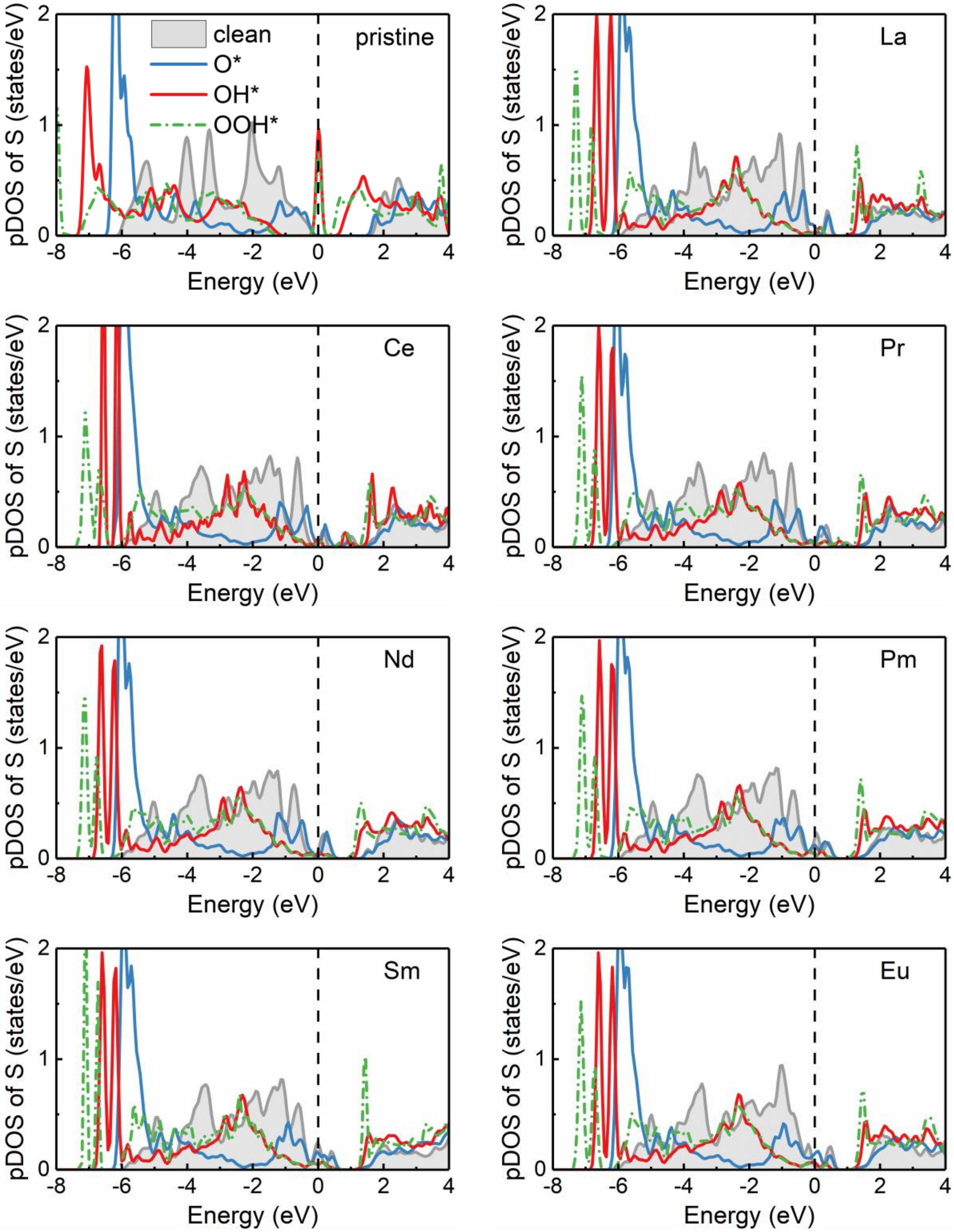
clean
O*
OH*
OOH*
pristine
La
Ce
Pr
Nd
Pm
Sm
Eu
pDOS of S (states/eV)
Energy (eV)

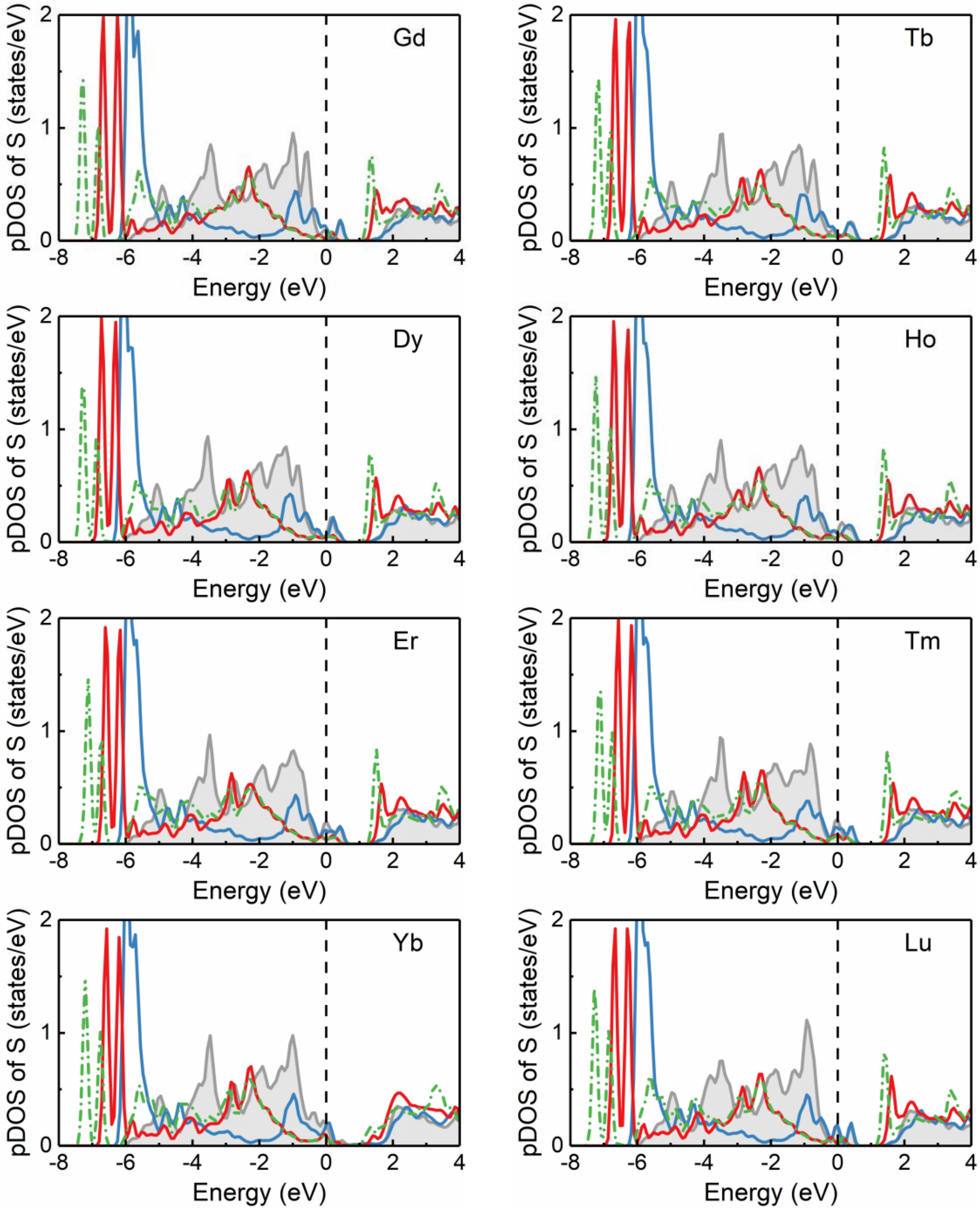

Figure S22. The pDOS spectra of the active S sites in pristine MoS_2 and Ln-MoS_2 before and after the adsorption of O, OH, and OOH. The highest occupied level is set to be 0 eV. Here, the chemical adsorption state of OOH on pristine MoS_2, not the more stable physical state, is chosen to calculate the pDOS, in order to reveal the creation of defect states induced by the chemical adsorption of OOH. Because the magnetism mechanism is not our focus here, the spin-up and spin-down parts are summed together for simplicity. Source data are provided as a Source Data file.

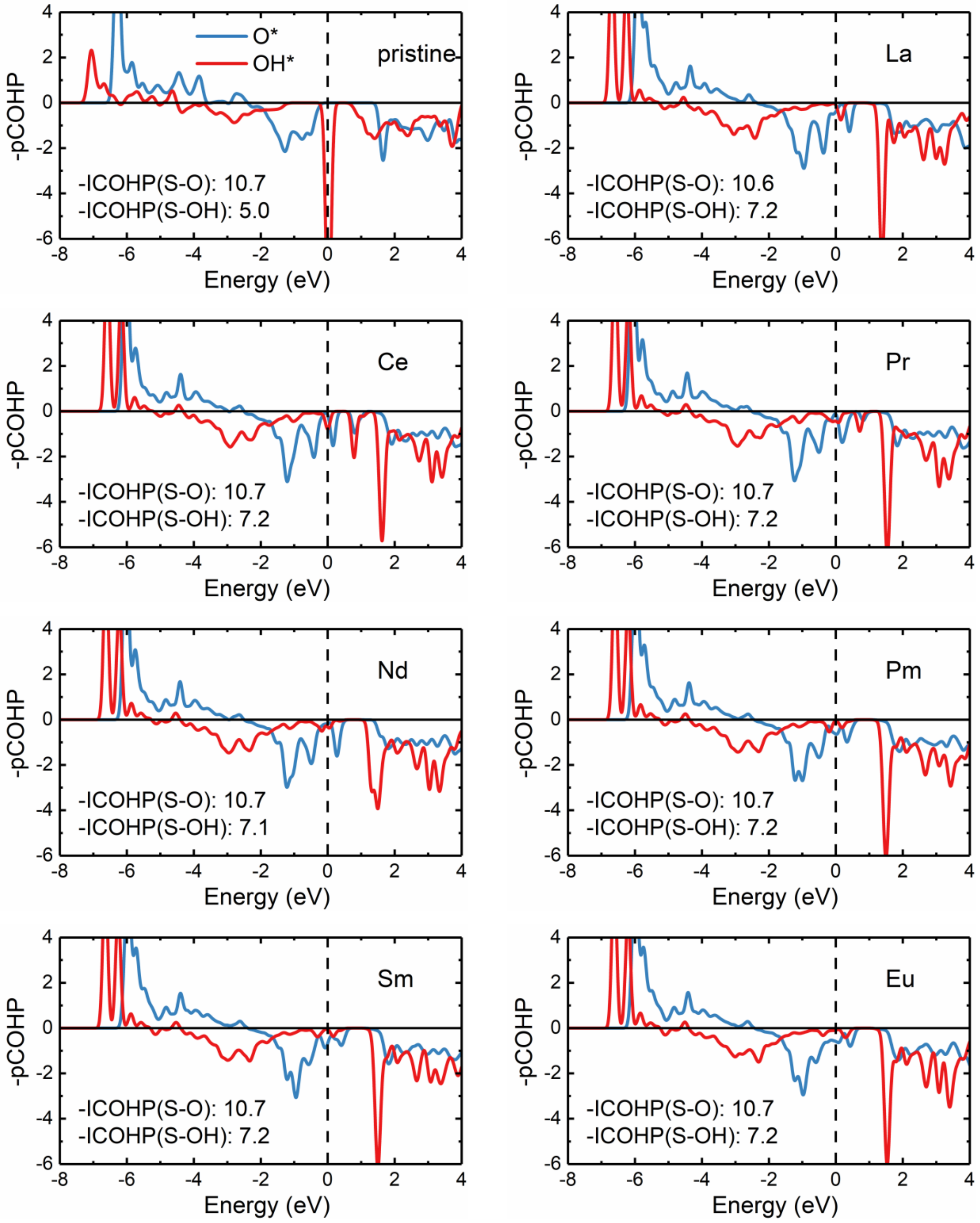
O*
OH*
pristine
-ICOHP(S-O): 10.7
-ICOHP(S-OH): 5.0
La
-ICOHP(S-O): 10.6
-ICOHP(S-OH): 7.2
Ce
-ICOHP(S-O): 10.7
-ICOHP(S-OH): 7.2
Pr
-ICOHP(S-O): 10.7
-ICOHP(S-OH): 7.2
Nd
-ICOHP(S-O): 10.7
-ICOHP(S-OH): 7.1
Pm
-ICOHP(S-O): 10.7
-ICOHP(S-OH): 7.2
Sm
-ICOHP(S-O): 10.7
-ICOHP(S-OH): 7.2
Eu
-ICOHP(S-O): 10.7
-ICOHP(S-OH): 7.2
-pCOHP
Energy (eV)

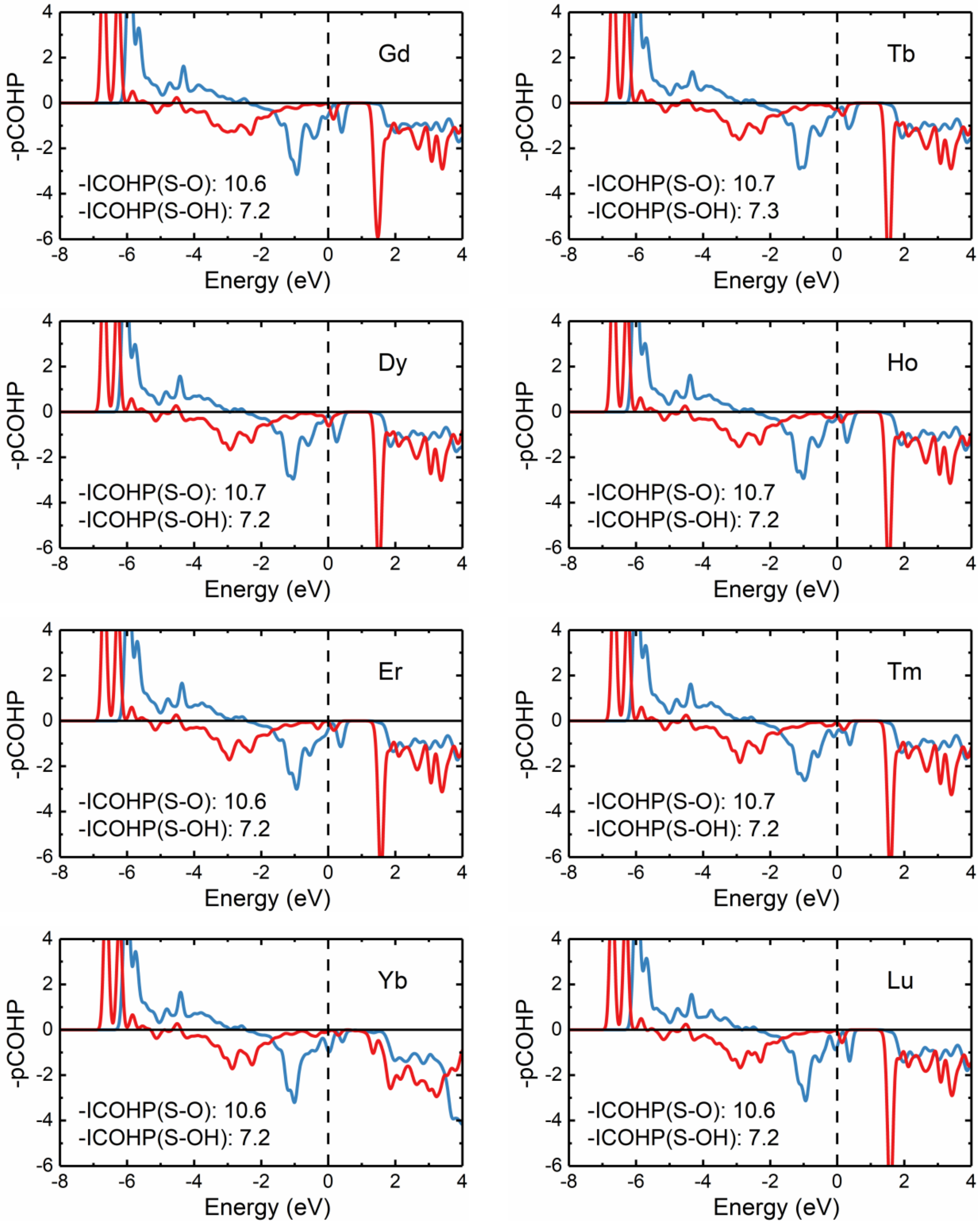

Figure S23. The -pCOHP spectra for the S-O bonds after O and OH adsorptions for pristine MoS_2 and Ln-MoS_2. The highest occupied level is set to be 0 eV and the spin-up and spin-down parts are summed together for simplicity. Source data are provided as a Source Data file.

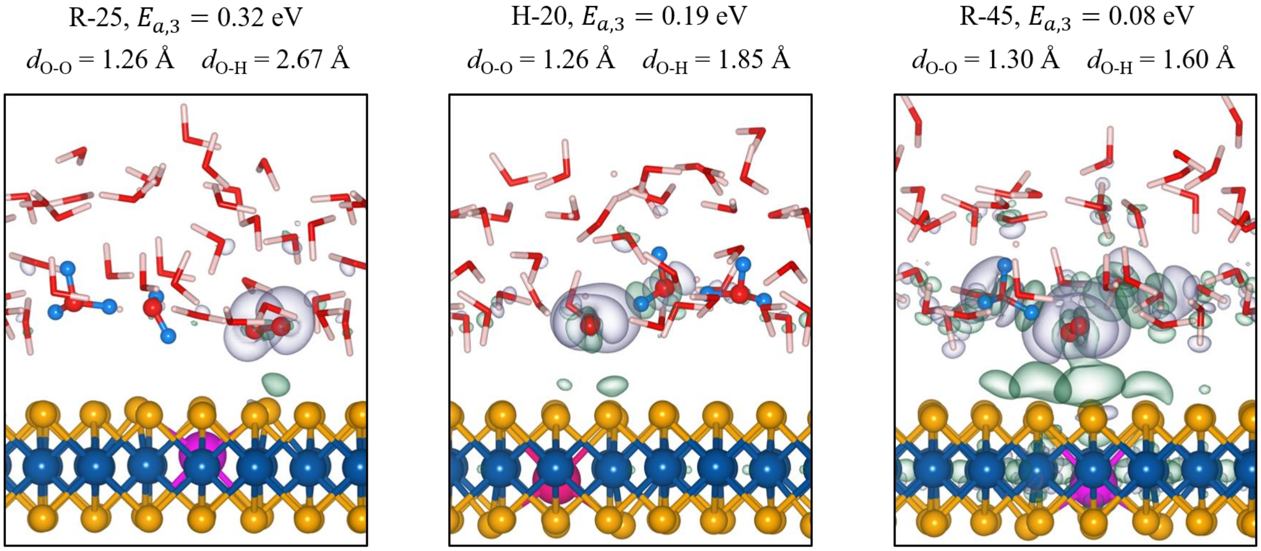

Figure S24. The differential electronic densities induced by O_2 adsorption with an additional H on Sm-MoS$_2$. The bond length of O_2^* ($d_{O\text{-}O}$) and the distance between O_2^* and its nearest H atom ($d_{O\text{-}H}$) are indicated. The electronic-density accumulation and depletion regions are shown in purple and green, respectively.

(I) Supplementary Details of the ORR Free Energy Diagrams for Ln-MoS_2

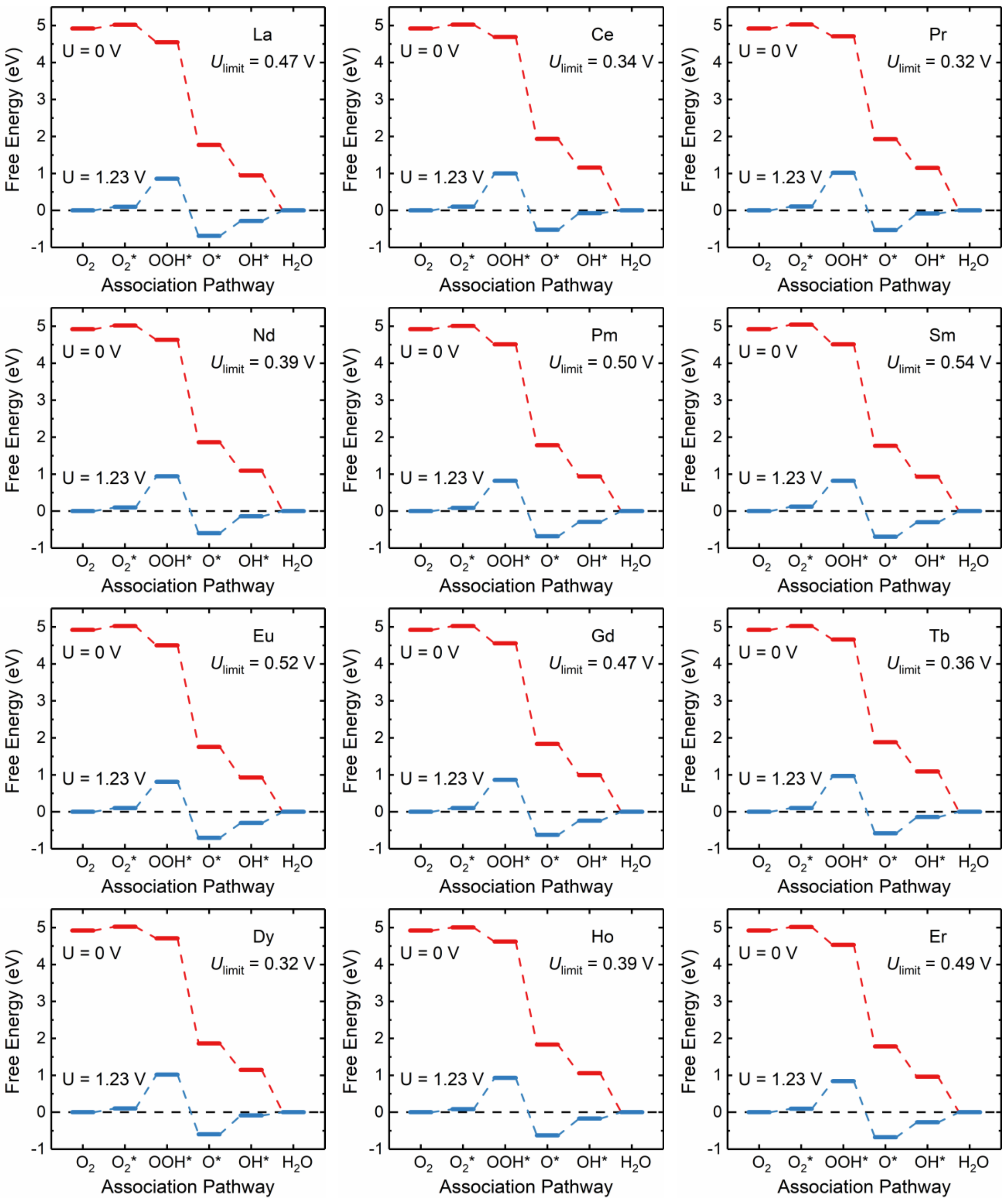

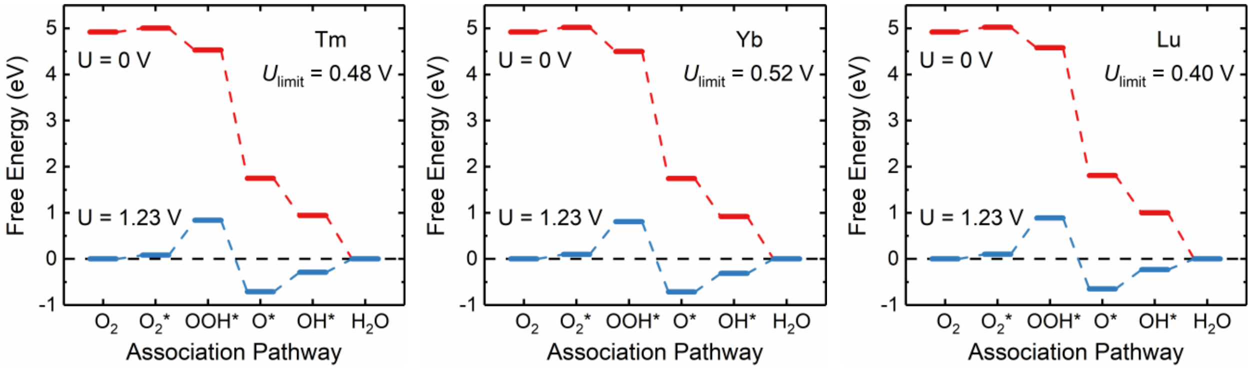

Figure S25. The free energy diagrams for the associative ORR pathway on Ln-MoS_2 with water effect. The electrode potential is set at 0 V and the ORR equilibrium potential of 1.23 V. The potential-limiting steps of Ln-MoS_2 all locate at the $O_2^* \rightarrow OOH^*$ step. Source data are provided as a Source Data file.

(J) Supplementary Formula and Results for Surface Pourbaix Diagrams

The chemical potentials (μ) of O^*, OH^*, and OOH^* are calculated based on the reaction equations of

$H_2O + * \rightarrow O^* + 2(H^+ + e^-)$,

$H_2O + * \rightarrow OH^* + H^+ + e^-$, and

$2H_2O + * \rightarrow OOH^* + 3(H^+ + e^-)$.

Taking $\mu(*)$ and $\mu(H_2O)$ as the references, the μ's of O^*, OH^*, and OOH^* can be expressed as

$$\mu(O^*) = G(O^*) - G(*) - G(H_2O) + G(H_2) - 2\times(0.059\text{pH} + |e|U_{\text{SHE}})$$
$$= \Delta G_{\text{ads}}(O^*) - 2\times(0.059\text{pH} + |e|U_{\text{SHE}}),$$

$$\mu(OH^*) = G(OH^*) - G(*) - G(H_2O) + 1/2G(H_2) - (0.059\text{pH} + |e|U_{\text{SHE}})$$
$$= \Delta G_{\text{ads}}(OH^*) - (0.059\text{pH} + |e|U_{\text{SHE}}),$$

$$\mu(OOH^*) = G(OOH^*) - G(*) - 2\times G(H_2O) + 3/2G(H_2) - 3\times(0.059\text{pH} + |e|U_{\text{SHE}})$$
$$= \Delta G_{\text{ads}}(OOH^*) - 3\times(0.059\text{pH} + |e|U_{\text{SHE}}),$$

where G is the Gibbs free energy of the corresponding surface state.

To examine the stability of Ln-MoS_2 in the electrochemical conditions, we additionally consider the possible loss of active S atoms (bonding with the Ln dopant) through the production of H_2S. The chemical potential of reaction associated with the corresponding reaction step ($* + 2(H^+ + e^-) \rightarrow V_S + H_2S$ (g)) is derived by

$$\mu(H_2S + V_S) = G(H_2S\ (g)) + G(V_S) - G(*) - G(H_2) + 2\times(0.059\text{pH} + |e|U_{\text{SHE}}),$$

which is used to define the chemical potential of the state of H_2S + S vacancy.

The simulated surface Pourbaix diagrams for all the fifteen Ln-MoS_2 surfaces are shown in **Figure S26**, and the corresponding μs of different surface states are shown in **Figure S27**. It can be clearly seen that any adsorbate is metastable around the U_{limit}, and only O^* will become stable at high enough potentials (above 0.87 ~ 0.97 V_{RHE}). The critical potential for the equilibria between O^* and clean surface ($U_{O^*/\text{clean}}$) and U_{limit} for all Ln-MoS_2 surfaces are summarized in **Figure S28**, where it can be seen that $U_{O^*/\text{clean}}$ is obviously higher than U_{limit} by 0.35~0.65 V, and both of them exhibit the reverse biperiodic chemical trends, simultaneously reflecting the same modulating trend in surface reactivity. Furthermore, $\mu(H_2S + V_S)$ keeps large positive values in the potential range of 0 ~ 1.23 V_{RHE}, indicating that the S atoms bonding with Ln dopant are stable against proton corrosion during the ORR process. It is also intriguing to observe the biperiodic chemical trend in $\mu(H_2S + V_S)$ (**Figure S29**), in accordance with the trend in Ln–S bond strength (i.e., reverse to the trend in E_f of Ln dopants, Figure 1c in the main text).

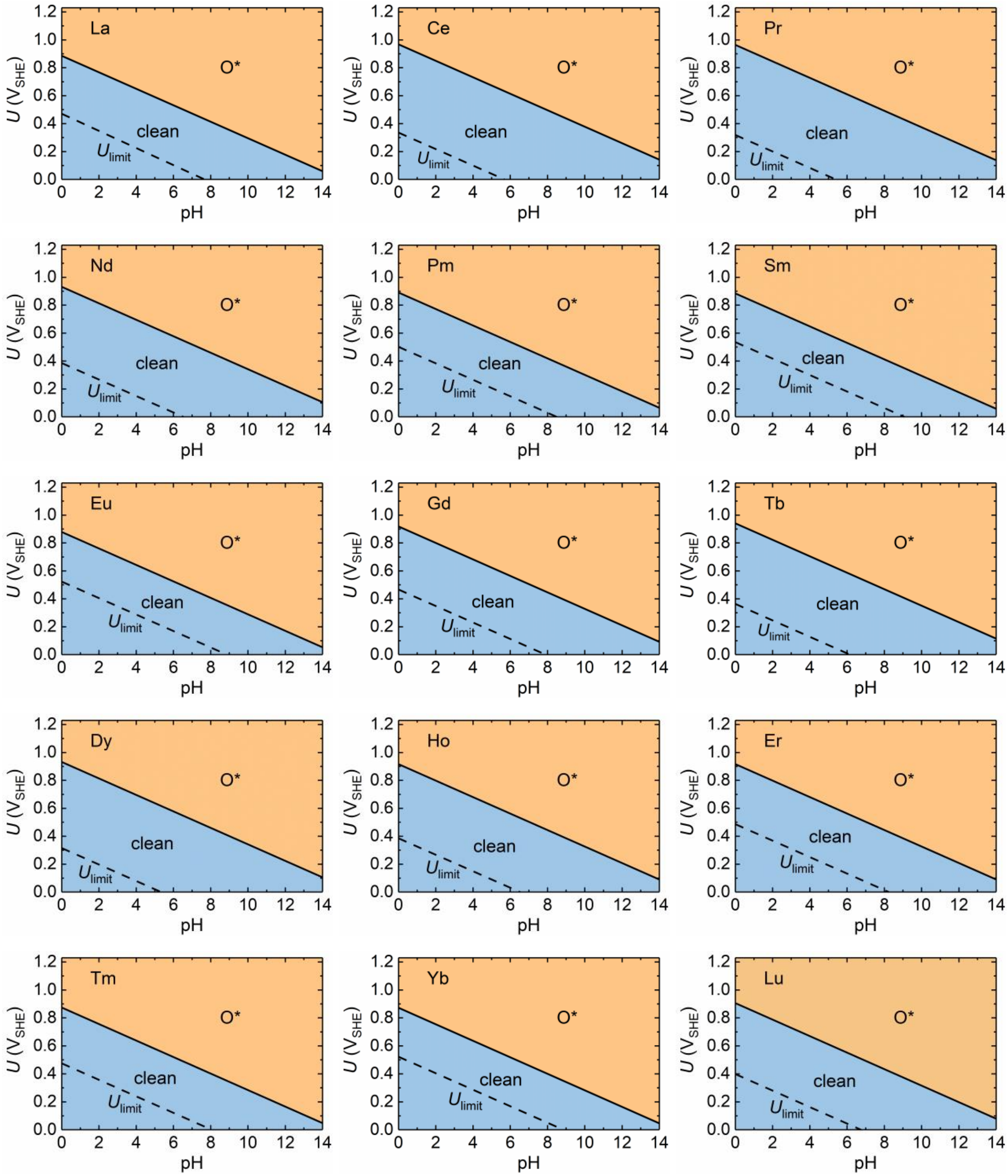

Figure S26. The surface Pourbaix diagrams of all the fifteen Ln-MoS$_2$ systems. Source data are provided as a Source Data file.

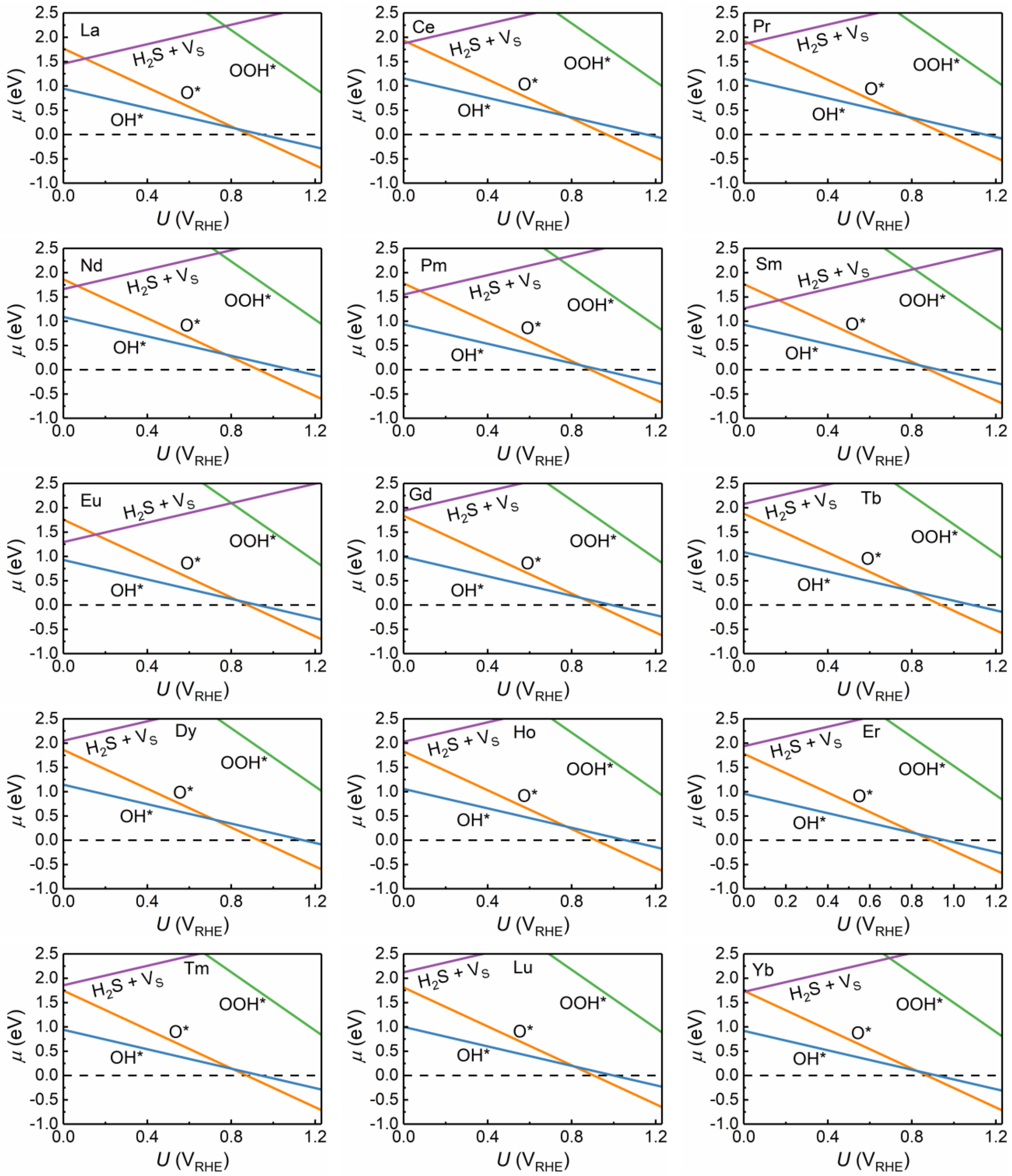

Figure S27. The μs of different surface states for all the fifteen Ln-MoS$_2$ systems. Source data are provided as a Source Data file.

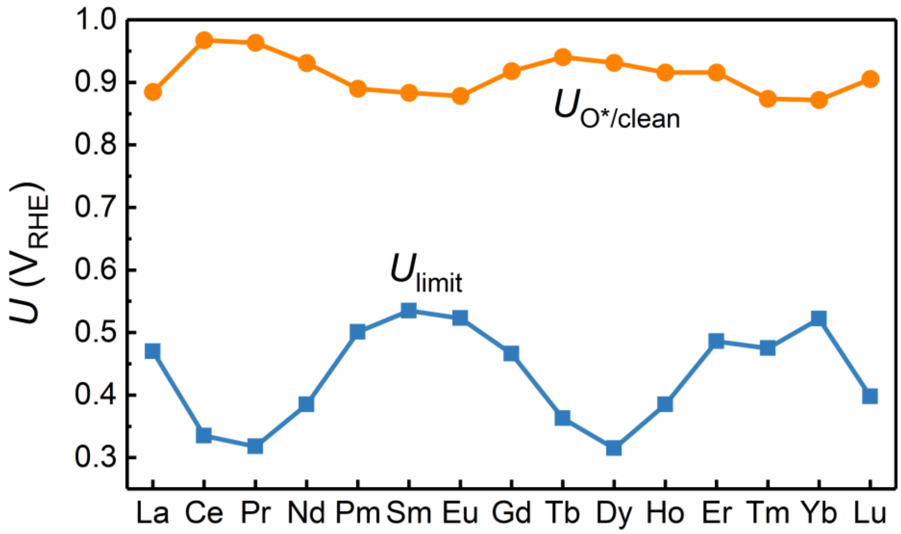

Figure S28. The variations of $U_{O*/clean}$ and U_{limit} with respect to Ln dopant type. Source data are provided as a Source Data file.

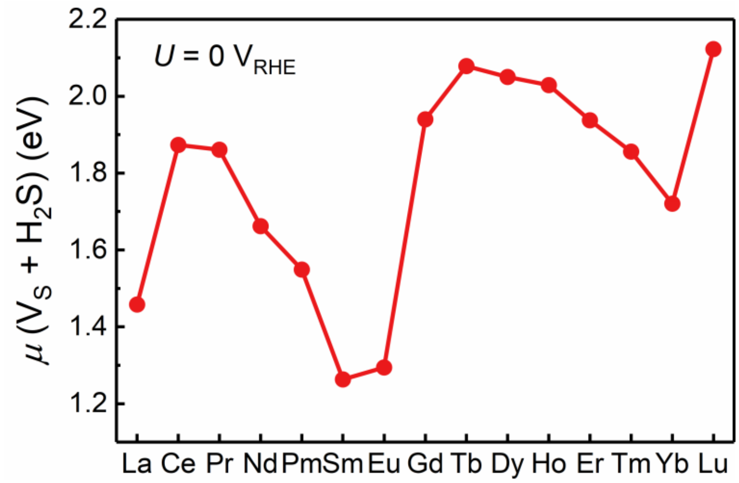

Figure S29. The $\mu(V_S + H_2S)$ for all the fifteen Ln-MoS_2 surfaces at 0 V_{RHE}. Source data are provided as a Source Data file.

(K) Supplementary Details for the Microkinetic Model

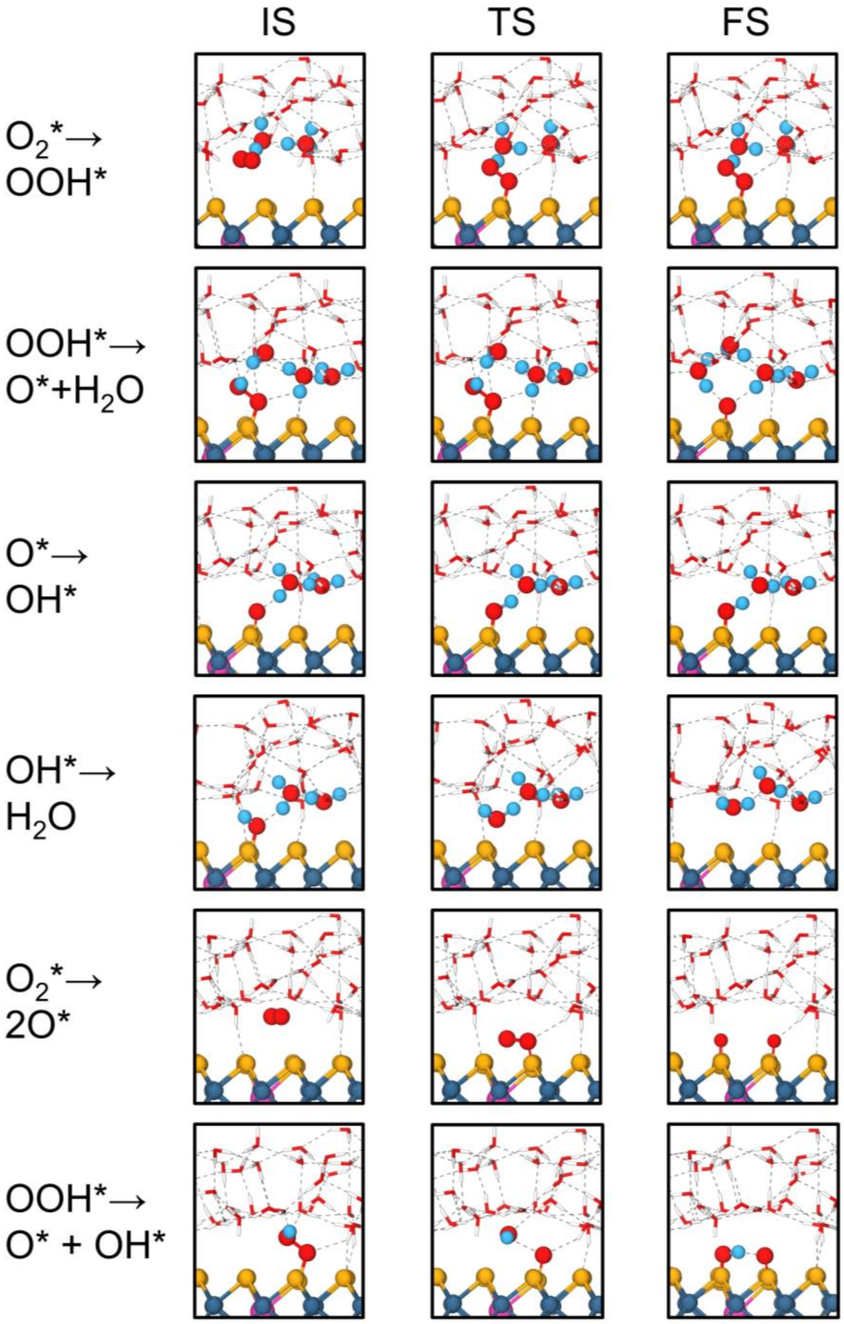

Figure S30. The atomic structures in the reaction paths of various ORR steps on the representative Sm-MoS$_2$ surface. The structures of initial states (IS), transition states (TS), and final states (FS) are shown and the optimal H-20 water configuration is choosen as the representative. The H atoms participating in the reactions are highlighted in light blue color.

Taking Sm-MoS_2 as a representative, the activation energy barriers ($E_{a,i}$) of all ORR steps are calculated with different water structures using the CI-NEB method [14], for which the results are shown in the main text (Figure 4a) and the atomic structures for different reaction paths are shown in **Figure S30**. It can be seen that the dissociation energy barrier of O_2^* is as high as 1.26 ~ 1.50 eV and then is difficult to overcome at room temperature, while the transition from O_2^* to OOH^* only requires a small energy barrier of 0.05~0.36 eV. Similarly, the $E_{a,10}$ of OOH^* dissociation (0.42~0.68 eV) is also considerably larger than that (0.01~0.03 eV) of the associative transition from OOH^* to O^*. Therefore, the ORR process on Ln-MoS_2 surface will take the OOH association pathway. Inspired by Hansen's work [15], the microkinetic model for the ORR process on Ln-MoS_2 surface can be constructed. The rate equations with the involved species (Eq. (1) ~ (6) in **Section D**) are given below:

$$\frac{\partial x_{O_2(dl)}}{\partial t} = k_1 x_{O_2(aq)} - k_{-1} x_{O_2(dl)} - k_2 x_{O_2(dl)} \theta_{*A} + k_{-2} \theta_{O_2^*A},$$

$$\frac{\partial \theta_{*A}}{\partial t} = k_6 \theta_{OH^*A} - k_{-6} \theta_{*A} x_{H_2O} - k_2 x_{O_2(dl)} \theta_{*A} + k_{-2} \theta_{O_2^*A},$$

$$\frac{\partial \theta_{O_2^*A}}{\partial t} = k_2 x_{O_2(dl)} \theta_{*A} - k_{-2} \theta_{O_2^*A} - k_3 \theta_{O_2^*A} - k_{-3} \theta_{OOH^*A},$$

$$\frac{\partial \theta_{OOH^*A}}{\partial t} = k_3 \theta_{O_2^*A} - k_{-3} \theta_{OOH^*A} - k_4 \theta_{OOH^*A} + k_{-4} x_{H_2O} \theta_{O^*A},$$

$$\frac{\partial \theta_{O^*A}}{\partial t} = k_4 \theta_{OOH^*A} - k_{-4} x_{H_2O} \theta_{O^*A} - k_5 \theta_{O^*A} + k_{-5} \theta_{OH^*A},$$

$$\frac{\partial \theta_{OH^*A}}{\partial t} = k_5 \theta_{O^*A} - k_{-5} \theta_{OH^*A} - k_6 \theta_{OH^*A} + k_{-6} \theta_{*A} x_{H_2O},$$

$$\theta_{*A} + \theta_{O_2^*A} + \theta_{OOH^*A} + \theta_{O^*A} + \theta_{OH^*A} = 1,$$

$$x_{O_2(aq)} = 2.34 \times 10^{-5},$$

$$x_{H_2O} = 1,$$

where θ is the coverage of ORR intermediates, t is time, and $x_{O_2(aq)}$ and $x_{O_2(dl)}$ are the mole fraction of O_2 in aqueous solution and electric double layer, respectively. These rate equations are solved at steady state, and then the turn over frequency and current density are finally calculated.

The rate constants k_1 and k_{-1} of the diffusion of O_2 molecule through the Nernstian diffusion layer on the rotating disk electrode (RDE) are calculated using [15]

$$k_1 = k_{-1} = \frac{D_{O_2} A c_{H_2O}}{2\delta}, \text{ and}$$

$$\delta = 1.61 D_{O_2}^{1/3} v^{1/6} \omega^{-1/2},$$

where D_{O_2} is the diffusivity for O_2, A is the average surface area of each active S site (contacting with 2 water molecules on average), c_{H_2O} is the bulk concentration of H_2O, δ is the thickness of the Nernstian diffusion layer, v is the kinematic viscosity of the electrolyte, and ω is the angular velocity of the RDE (in rad/s). There are three active surface S sites close to the Ln dopant in MoS_2, i.e., three active sites associated with one Ln dopant, thus $A = A_0 / 3$, where A_0 is the surface area of the Ln-MoS_2 supercell constructed in this work. We only consider the properties of monomer-Ln dopants, because the Ln dopants in the experimental samples have a good dispersion with the inter-dopant distances of ~20 Å [1, 16].

According to the transition-state theory, k_2 for the adsorption process of O_2 is calculated by

$$k_2 = \frac{k_B T}{h} \exp(-\frac{G_{a,2}}{k_B T}),$$

where k_B is Boltzmann constant, T is Temperature, h is Planck constant, and $G_{a,2}$ is the activation free energy. Due to the weak adsorption of O_2 on Ln-MoS_2, $G_{a,2}$ is manly contributed by the reorientation effect of solvent molecules, which is calculated to be 0.07 ~ 0.17 eV (see **Figure S16** and Figure 2c in the main text) here. It has a higher magnitude (0.22 ~ 0.28 eV) on Pt [15] due to the stronger water adhesion. In our testing calculations using $G_{a,2}$ from 0.07 to 0.28 eV, U_{half} has negligible variation (<0.002 V, **Figure S31**), because $O_2(dl) \rightarrow O_2^*$ is not the ORR-limiting step (see main text, Figure 4e).

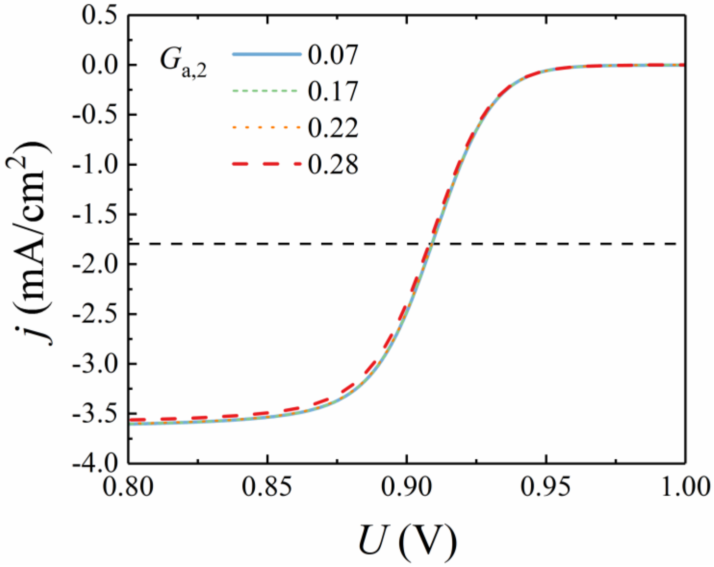

Figure S31. The simulated polarization curves for Sm-MoS_2 with different $G_{a,2}$'s. Source data are provided as a Source Data file.

The k_i of the i'th electrochemical step is calculated by

$$k_i = \frac{k_B T}{h} \exp\left(-\frac{G^0_{a,\,i}}{k_B T}\right) \cdot \exp\left[-\frac{e\alpha_i(U - U^0_i)}{k_B T}\right],$$

where $G^0_{a,i}$ is the activation free energy at the equilibrium potential (U^0_i) of the i'th step, α_i is the transfer coefficient (set to be 0.5), and U is the electrode potential. U^0_i is calculated by U^0_i = -ΔG^0_i/e, where ΔG^0_i is the free-energy change of the i'th reaction step at 0 V. The reverse-reaction rate constant $k_{\text{-}i}$ is calculated by exploiting the equilibrium constant (K_i):

$$k_{\text{-}i} = \frac{k_i}{K_i},$$

$$K_i = \exp\left(\text{-}\frac{\Delta G_i}{k_B T}\right).$$

The current density (j) is calculated by

$$j = 4e{\cdot}\rho{\cdot}\mathrm{TOF}_{O_2},$$

where 4 is the number of transferred electrons, ρ is the surface density of active sites, and TOF_{O_2} is the turnover frequency of O_2. The kinetic parameters with constant values are summarized in **Table S1**.

Table S1. The constant kinetic parameters used for the microkinetic simulations at 25 □.

Parameter	Value
Temperature (T)	298.15 K
Boltzmann constant (K_B)	1.38×10^{-23} J/K
Planck constant (h)	4.14×10^{-15} eV s
Rotation rate (ω)	1600 rpm
Bulk concentration of H_2O (c_{H_2O})	55.6 mol/L
Kinematic viscosity of H_2O (v)	1.009×10^{-6} m^2 s^{-1}
Diffusion coefficient of O_2 (D_{O_2})	2.42×10^{-5} cm^2 s^{-1}

Table S2. The temperature correction ($\Delta\varepsilon_{zpe}$ - $T\Delta s$) of $E_{a,i}$ of Sm-MoS_2 at 25 □.

ORR steps	$\Delta\varepsilon_{zpe}$ - $T\Delta s$
$O_2^* \rightarrow OOH^*$	0.002
$OOH^* \rightarrow O^*$	-0.018
$O^* \rightarrow OH^*$	-0.038
$OH^* \rightarrow H_2O$	-0.084

Due to a very low diffusion barrier (~ 0.09 eV) [17], protons can quickly reach the vicinity of the adsorbates. We choose the representative H-20 water environment, and calculate the activation

energies $E_{a,i}$ for many selected Ln-MoS_2 (Ln = La, Ce, Pr, Sm, Dy, Yb, and Lu). The temperature corrections (**Section E**) for the $E_{a,i}$'s of Sm-MoS_2 at 25 °C (**Table S2**) are negative or only 0.002 eV. Thus, we can safely assume $G^0_{a,\,i} \approx E_{a,\,i}$. The Brønsted-Evans-Polanyi relationship [15] for the $O_2^* \rightarrow OOH^*$ step is shown in the main text (Figure 4b), which can be used to estimate the $E_{a,3}$'s of other Ln-MoS_2 systems when having calculated the corresponding reaction energies (ΔE_3). Due to the important role of this step in determine the polarization curves (see Figure 4e in the main text), we further investigate the structural mechanism underlying the $E_{a,3}$ with the example of Sm-MoS_2. In **Figure S24** and **S32a**, we can see that different water configurations will result in different distances between O_2^* and its nearest H atom ($d_{\text{O-H}}$), which $E_{a,3}$ closely correlates with, and the average $E_{a,3}$ is 0.19 eV. We further perform an AIMD simulation (300 K, 40 ps, 0.5 fs/step) on the structure of O_2^* with a H atom nearby, the variation of $d_{\text{O-H}}$ (**Figure S32b**) clearly shows that apart from freely moving within the water layer in most of the time, the H neighbor frequently gets close to the O_2 molecule (1.8~2.0 Å), which can facilitate the following protonation reaction of O_2^*. Thus, the Sm-MoS_2/water interface with a $d_{\text{O-H}}$ around 2 Å (e.g., 1.86 Å) should be chosen to derive an appropriate $E_{a,3}$, and a too short (e.g., 1.6 Å) or too long (e.g., 2.7 Å) will result in an underestimated or overestimated reaction barrier (**Figure S32a**). Furthermore, although the adsorption of O_2^* on Sm-MoS_2 surface is relatively weak, it does not escape from the Sm-MoS_2/water interface during the AIMD simulation, indicating its preferred stability to act as an initial surface reactant for the whole highly-active ORR process.

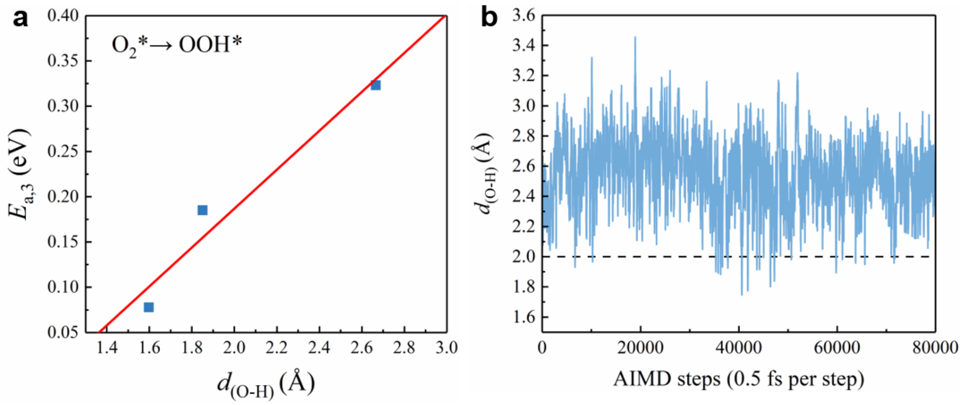

Figure S32. The $E_{a,\,3}$–$d_{\text{O-H}}$ relationship and the variation of $d_{\text{O-H}}$ in the AIMD simulation. (a) The relationship between $E_{a,\,3}$ and $d_{\text{O-H}}$ for Sm-MoS_2 surface under the three considered water structures. (b) The variation of $d_{\text{O–H}}$ in the AIMD simulation at 300 K for Sm-MoS_2/water interface with an O_2^* on surface and an additional H atom in water. Source data are provided as a Source Data file.

For the $OOH^* \rightarrow O^*$ step, the $E_{a,4}$ of Sm-MoS_2 is lower than 0.03 eV as shown in the main text (Figure 4a) and the U_4^0 is around 2.72 V, indicating that this step will proceed spontaneously when the electrode potential is lower than 1.23 V. Thus, we can safely assume that the $E_{a,4}$'s for other Ln-MoS_2 surfaces all equal 0 eV. For the $O^* \rightarrow OH^*$ step, the calculated $E_{a,5}$'s of La-, Ce-, Pr-, Sm-, Dy-, and Yb-MoS_2 are very close to each other around 0.12 eV (**Figure S33a**), except for Lu-MoS_2 (~ 0.09 eV), thus 0.12 eV is adopted for other Ln-MoS_2 surfaces. The Brønsted-Evans-Polanyi relationship [15] for the $E_{a,6}$ ($OH^* \rightarrow H_2O$) values is also shown in **Figure S33b**. Although there is a certain deviation of the as-calculated data from this linear relationship, the total ORR reaction rate has a very small dependence on $E_{a,6}$ (i.e., having a $X_{RC,6}$ close to 0, see Figure 4e in the main text). Therefore, a variation of 0.1 ~ 0.4 eV in $E_{a,6}$ will cause negligible change in the reaction rate. The simulated current-potential polarization curves for all the Ln-MoS_2 systems are shown in **Figure S34**.

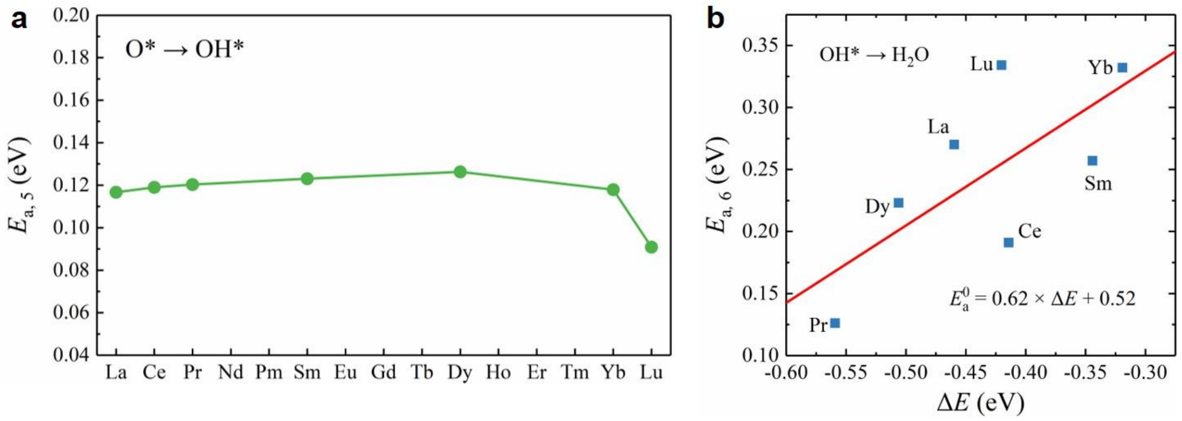

Figure S33. The variation trends of the activation energies for the $O^* \rightarrow OH^*$ ($E_{a,5}$) and $OH^* \rightarrow H_2O$ ($E_{a,6}$) reaction steps. (a) The variation of $E_{a,5}$ with respect to the Ln type and (b) the linear relationship between $E_{a,6}$ and its corresponding reaction energy (ΔE_6), where the La-, Ce-, Pr-, Sm-, Dy-, Yb- and Lu-MoS_2 are chosen as the representatives. Source data are provided as a Source Data file.

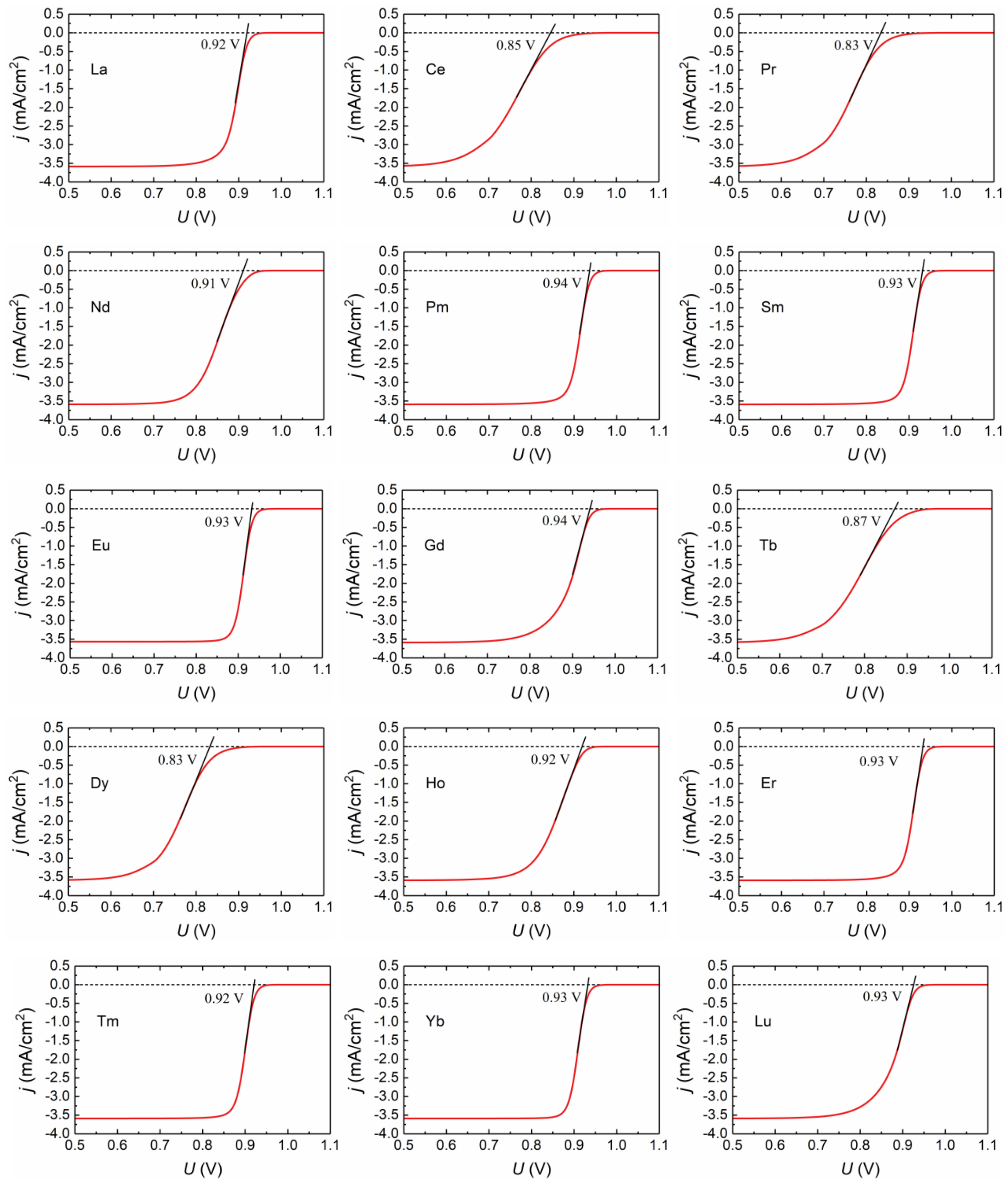

Figure S34. Simulated polarization curves for all the Ln-MoS_2 surfaces at 1600 rpm rotation speed and 25 □. The onset potentials are labeled, which are calculated as the intersection of the tangent at the inflection point with the horizontal zero-current line. Source data are provided as a Source Data file.

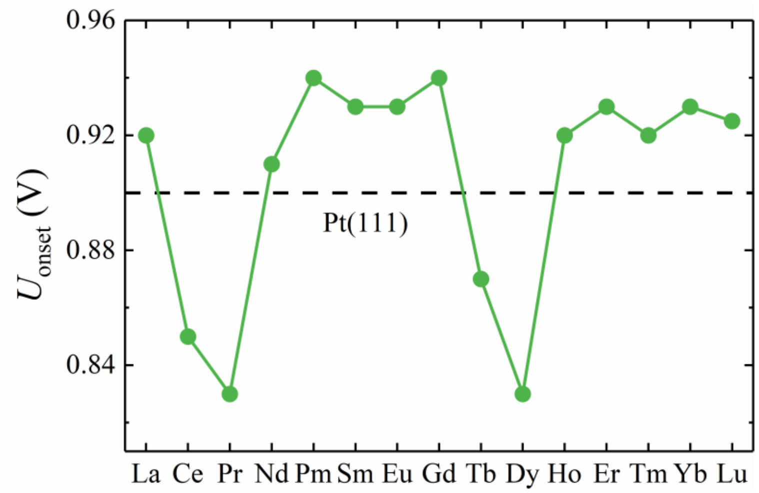

Figure S35. The onset potentials (U_{onset}) for Ln-MoS_2 surfaces. Source data are provided as a Source Data file.

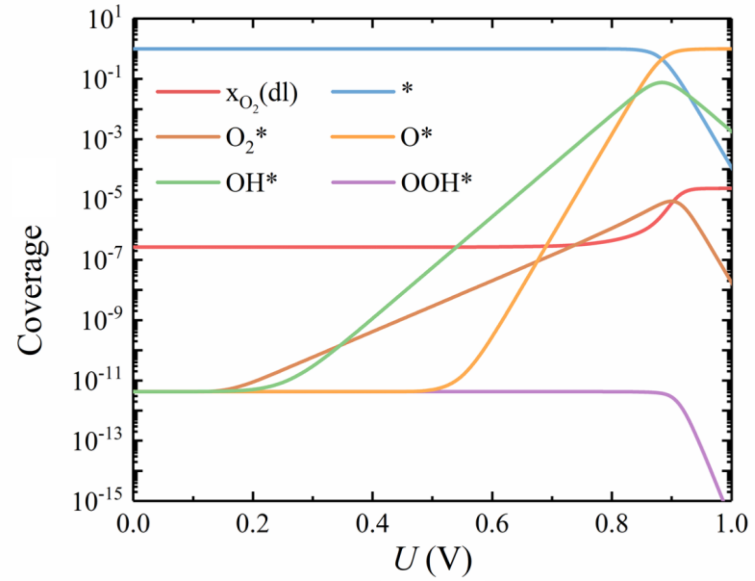

Figure S36. The potential dependences of ORR-intermediate coverages and $x_{O_2(dl)}$ on Sm-MoS_2. Source data are provided as a Source Data file.

The effects of RDE rotation speed (within a typical range from 900 to 3200 rpm) and temperature (within a typical range from 25 to 60 □) on the simulated polarization curves are evaluated here (**Figure S37**), where the representative Sm-MoS_2 system is used. Both the rotation speed and temperature will affect the current density mainly through influencing the diffusion of O_2 from the bulk electrolyte to the material-electrolyte interface. It can also be seen from **Figure S37** that although they can bring obvious change in the absolute value of current density at surface potential of ≤ 0.9 V, they only slightly shift the U_{half} by ~ 0.01 V.

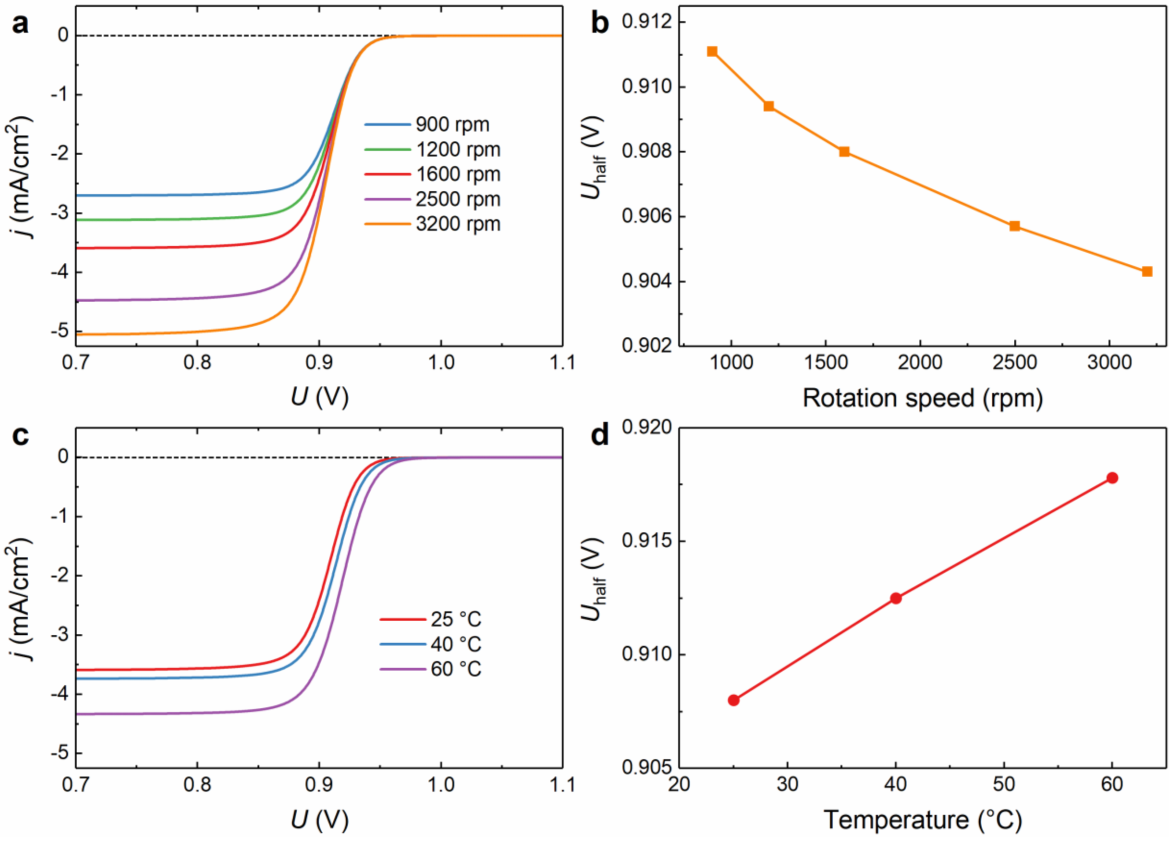

Figure S37. The simulated polarization curves and corresponding half-wave potentials for Sm-MoS_2 at different rotation speeds and temperatures. (a) and (b) reflect the rotation speed effect. (c) and (d) reflect the temperature effect. Source data are provided as a Source Data file.

The above observations on the effects of RDE rotation speed and temperature actually are quite consistent in essence with the kinetic rate control analysis in the main text (Figure 4e). The effect of temperature can be further decomposed into reducing the initial concentration of O_2 in water (x_{O_2}(aq), from 2.45×10^{-5} at 25 □ to 1.58×10^{-5} at 60 □ [18]) and increasing the reaction rate constant (**Figure S38**). The temperature dependence of k_1 and k_{-1} mainly originate from two factors (also see the formula for microkinetic model above): (1) the increase of O_2 diffusion coefficient D_{O_2} from 2.4×10^{-5} at 25 □ to 4.6×10^{-5} cm^2 s^{-1} at 60 □, and (2) the decrease in water kinematic viscosity v from 1.01×10^{-6} at 25 □ to 0.47×10^{-6} m^2 s^{-1} at 60 □ [18]. Apart from k_1 and k_{-1}, the reaction rates for the transitions between ORR intermediates at different temperatures are derived from the transition-state theory (see the formula for k_i above). In **Figure S38**, the dependence of current density on the individual thermal changes of rate constant and O_2 concentration is quantitatively disentangled. In addition, the effect of RDE rotation speed is attributed to the changed Nernstian diffusion layer and the associated changes in k_1 (see its formula above).

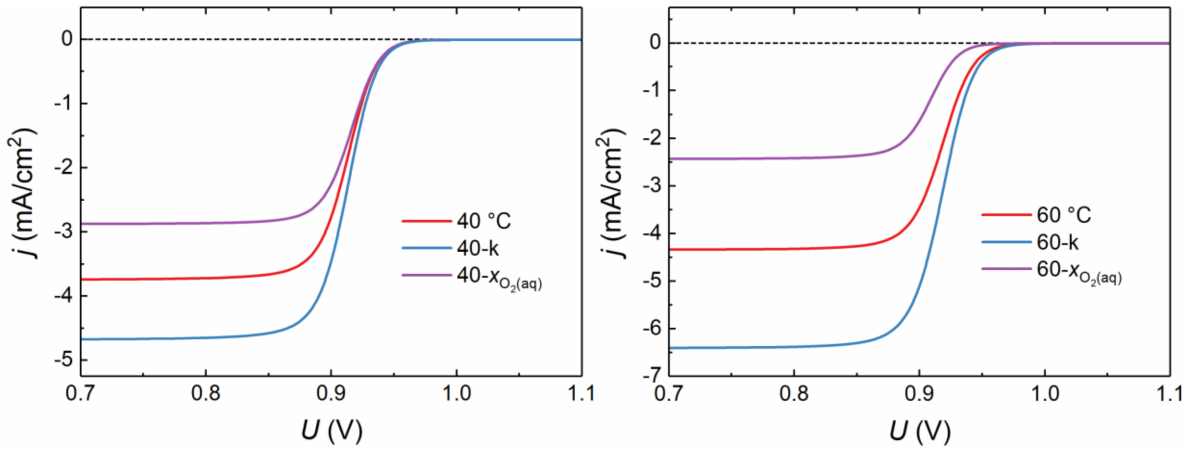

Figure S38. The thermal-effect mechanism disentangling for the polarization curve of the representative Sm-MoS_2 system. The temperatures of 40 and 60 □ are considered, and the curves denoted with keys of "40 □" ("60 □") , "40-k" ("60-k"), and "40-$x_{O_2(aq)}$" ("60-$x_{O_2(aq)}$") correspond to the simulated overall curves, the curves simulated only with the rate constants changed in accordance with temperature, and the curves simulated only with $x_{O_2(aq)}$ changed, respectively. Source data are provided as a Source Data file.

Table S3. Summary of the ORR activities of different metal surfaces reported in literature. The current densities are measured in experiments at 0.9 V, and the ΔG_{ads}(OH)s are calculated by DFT.

Materials	j / j_{Pt}	$\Delta G_{ads}(OH) - \Delta G_{ads}(OH)^{Pt}$ (eV)	Experimental conditions
Pt (111)[a,] [12, 19]	1	0	1600 rpm, 0.1 M $HClO_4$, 23 ± 2 □
Pt (100) [20, 21]	0.405	-0.15	1600 rpm, 0.1 M $HClO_4$, room temperature
Ag (100) [21, 22]	0.001	-0.2	1600 rpm, 0.1 M KOH, 20 □
Pd (111) [12, 23]	0.135	-0.13	1600 rpm, 0.1 M $HClO_4$, room temperature
Au (111) [12, 24]	0.035	0.44	2500 rpm, 0.1 M KOH, 20 □
Au (100) [21, 24]	0.682	0.35	2500 rpm, 0.1 M KOH, 20 □
Pt_3Ti[b,] [25]	1.342	0.185	1600 rpm, 0.1 M $HClO_4$, 60 □
Pt_3Fe [25]	2.544	0.155	1600 rpm, 0.1 M $HClO_4$, 60 □
Pt_3Co [25]	2.919	0.145	1600 rpm, 0.1 M $HClO_4$, 60 □
Pt_3Ni [25]	2.215	0.135	1600 rpm, 0.1 M $HClO_4$, 60 □
Pt_3Ni (111) [26]	3.321	0.1	1600 rpm, 0.1 M $HClO_4$, 60 □
Pt_3Y [19]	2.967	0.15	1600 rpm, 0.1 M $HClO_4$, 23 ± 2 □
Pt_3Sc [19]	1.35	0.15	1600 rpm, 0.1 M $HClO_4$, 23 ± 2 □
Pt/Ir(111)[c,] [27, 28]	0.317	0.19	1600 rpm, 0.1 M $HClO_4$, room temperature
Pt/Au(111) [27, 28]	0.709	-0.08	1600 rpm, 0.1 M $HClO_4$, room temperature
Pt/Pd(111) [27, 28]	1.27	0.1	1600 rpm, 0.1 M $HClO_4$, room temperature
Pt/Rh(111) [27, 28]	0.428	0.21	1600 rpm, 0.1 M $HClO_4$, room temperature

a. According to Ref. [12, 15, 19], j at 0.9 V and ΔG_{ads}(OH) of Pt(111) are taken as 1.2 mA/cm^2 and 0.8 eV to compare with that of Ln-MoS_2, respectively.

b. Pt_3X (X = Ti, Fe, Co, Ni) is the polycrystalline alloy annealed in ultrahigh vacuum, where the ΔG_{ads}(OH) is estimated from ΔG_{ads}(O), i.e. $\Delta G_{ads}(OH) - \Delta G_{ads}(OH)^{Pt} \approx 0.5[\Delta G_{ads}(O) - \Delta G_{ads}(O)^{Pt}]$ [25, 29].

c. Pt/X(111) (X = Ir, Au, Pd) is the platinum monolayer supported on X(111) surface. The ΔG_{ads}(OH) of Pt/Rh(111) is estimated from ΔG_{ads}(O), i.e. $\Delta G_{ads}(OH) - \Delta G_{ads}(OH)^{Pt} \approx 0.31[\Delta G_{ads}(O) - \Delta G_{ads}(O)^{Pt}]$ [27, 28].

(L) Supplementary Numerical Tests Validating the DFT Accuracy

When dealing with Ln elements that have highly localized 4*f* orbitals, there is the *electronic self-interaction problem* that we need to concern for the conventional PBE-GGA functional [30]. We use the efficient *DFT plus Hubbard U* method [31] to probe the influence of this problem on the accuracy of calculated surface reactivity. The tested adsorption free energies (ΔG_{ads}) of O and OH adsorbates on the active S sites of Ce-MoS_2 and Gd-MoS_2, which respectively have the minimum and maximum magnetic moments (see **Figure S7c**), are found nearly independent on the U_{eff} value (energy variation <0.03 eV, see **Table S4**). Furthermore, the effect of spin-orbit coupling (SOC) for the 4*f* orbitals is also tested, and its influence on the ΔG_{ads}s of adsorbates on active S site is also negligible (energy variation <0.01 eV, see **Table S5**). The negligible effects of both U_{eff} and SOC acting on the Ln-4*f* electrons are understandable, because the adsorption of ORR intermediates resides on S atom, for which the regular PBE-GGA functional is accurate. It should be the same accuracy reason for many successful computational studies on MoS_2-based systems using the PBE-GGA functional, which have also been closely verified in experiment. For example, Deng et al. screen the hydrogen evolution reaction behavior of many transition metal doped MoS_2 and find the optimal activity of Pt-MoS_2 [32]; Li et al. activate and optimize the hydrogen evolution activity of MoS_2 surface with vacancy concentration and strain [33]; Tsai et al. reveal the electrochemical desulfurization potential of MoS_2 for generating S-vacancies on basal plane [34]. It is an indirect way for the Ln dopants to affect the ORR activity of MoS_2 by changing the Ln–S bond character, and the 4*f* orbitals on Ln atom will hybridize with the higher-energy orbitals (e.g., 5*d*, 6*s*, and 6*p*), which contribute to the bond character and strength. The convergence testing calculations for ΔG_{ads}s using different cutoff energies and reciprocal-point mesh sizes are shown in **Table S6** and **S7**, which clearly show that the settings used in this work (450 eV and 2×2×1) are stringent enough to yield accurate ΔG_{ads}s (energy convergence < 0.01 eV).

Table S4. The ΔG_{ads}(O) and ΔG_{ads}(OH) on Ce-MoS_2 and Gd-MoS_2 with the effective on-site Hubbard correlation parameter (U_{eff}). The range of U_{eff} is from 0.0 to 6.0 eV.

U_{eff} (eV) / ΔG_{ads} (eV)	0.0	1.0	2.0	3.0	4.0	5.0	6.0
Ce-MoS_2 ΔG_{ads} (O)	1.83	1.84	1.84	1.85	1.85	1.85	1.86
Ce-MoS_2 ΔG_{ads} (OH)	1.43	1.43	1.44	1.44	1.44	1.45	1.45
Gd-MoS_2 ΔG_{ads} (O)	1.75	1.75	1.75	1.75	1.75	1.75	1.75
Gd-MoS_2 ΔG_{ads} (OH)	1.29	1.29	1.30	1.30	1.30	1.30	1.30

Table S5. The ΔG_{ads}(O) and ΔG_{ads}(OH) on Ce-MoS_2 and Gd-MoS_2 with and without the spin-orbit coupling effect.

ΔG_{ads} (eV)	Without SOC	With SOC
Ce-MoS_2 ΔG_{ads} (O)	1.83	1.83
Ce-MoS_2 ΔG_{ads} (OH)	1.43	1.43
Gd-MoS_2 ΔG_{ads} (O)	1.75	1.75
Gd-MoS_2 ΔG_{ads} (OH)	1.29	1.30

Table S6. The convergence tests using different cutoff energies for the ΔG_{ads}(O) and ΔG_{ads}(OH) on Ce-MoS_2 and Gd-MoS_2.

Cutoff energy (eV) / ΔG_{ads} (eV)	450	500	600
Ce-MoS_2 ΔG_{ads} (O)	1.83	1.84	1.83
Ce-MoS_2 ΔG_{ads} (OH)	1.43	1.43	1.42
Gd-MoS_2 ΔG_{ads} (O)	1.75	1.75	1.75
Gd-MoS_2 ΔG_{ads} (OH)	1.29	1.29	1.29

Table S7. The convergence tests using different reciprocal-point mesh sizes for the ΔG_{ads}(O) and ΔG_{ads}(OH) on Ce-MoS_2 and Gd-MoS_2.

k point / ΔG_{ads} (eV)	2×2×1	3×3×1	4×4×1
Ce-MoS_2 ΔG_{ads} (O)	1.83	1.83	1.83
Ce-MoS_2 ΔG_{ads} (OH)	1.43	1.43	1.43
Gd-MoS_2 ΔG_{ads} (O)	1.75	1.76	1.75
Gd-MoS_2 ΔG_{ads} (OH)	1.29	1.28	1.27

Regarding the structural model for Ln-MoS_2, it is the monomer-Ln dopants observed in experimental samples (see Figure 1b in main text), where the inter-dopant distances are ~20 Å with a good dispersion. Therefore, such monomer dopant model is considered here for the study of ORR activity. To reveal the effect of Ln concentration on surface reactivity, the supercell-size effect on ΔG_{ads} is calculated (**Table S8**), which shows that an energy convergence of <0.02 eV can be achieved by the supercell of $4\times2\sqrt{3}$ used in this work. The weak supercell-size effect on Ln dopant can be also well reflected by the supercell-size dependence of E_f (**Figure S39** and **S40**), and the supercell used in this work can guarantee an energy convergence of 0.1~0.2 eV (i.e., < 3%), and the biperiodic chemical trend in E_f has no obvious quantitative change when using a larger supercell (e.g., $6\times2\sqrt{3}$, **Figure S40**). Furthermore, the very weak magnetic coupling strength between two

neighboring Ln dopants (**Table S9**) in the used supercell additionally proves the negligible inter-dopant interaction. In the calculation of magnetic couplings, the two Gd dopants are constructed by doubling the supercell along one direction (designated as the *x* direction here), and the relative stability between the antiferromagnetic (AFM) and ferromagnetic (FM) configurations are calculated. The AFM−FM energy difference is found to be as small as −0.001 ~ −0.007 eV for supercells ranging from 2×3×2√3 to 2×5×2√3, which clearly indicates that neighboring Ln dopants have the paramagnetic coupling state (i.e., AFM≈FM), and the spin polarization of a Ln dopant can be individually treated in DFT calculations. Furthermore, the ORR active site actually resides on the Ln-bonded S atom, which has a nonmagnetic character. This means that the inter-dopant magnetic coupling state is not only very weak, but also intuitively irrelevant to the ORR activity. This situation is different from that of transition-metal doped nitrogenated graphene [35, 36], where the active sites exactly reside on the transition-metal dopants and their magnetic coupling effect may play a role in the ORR activity. Therefore, according to the above comprehensive testing calculations for supercell-size effects, the structure model for Ln-MoS_2 as used in this work (i.e., a Ln dopant in the 4×2√3 supercell) can well represent the realistic Ln-MoS_2 samples with a high numerical accuracy.

Table S8. The tested supercell-size effect on ΔG_{ads}(O) and ΔG_{ads}(OH) on Ce-MoS_2 and Gd-MoS_2.

Supercell / ΔG_{ads} (eV)	4×2√3	6×3√3	8×4√3	10×5√3
Ce-MoS_2 ΔG_{ads} (O)	1.83	1.84	1.84	1.84
Ce-MoS_2 ΔG_{ads} (OH)	1.43	1.41	1.40	1.41
Gd-MoS_2 ΔG_{ads} (O)	1.75	1.74	1.75	1.75
Gd-MoS_2 ΔG_{ads} (OH)	1.29	1.29	1.32	1.31

Table S9. The energy difference between the antiferromagnetic (AFM) and ferromagnetic (FM) configurations for two neighboring Gd dopants in MoS_2 supercells.

Supercell / AFM-FM	2×2×2√3	2×3×2√3	2×4×2√3	2×5×2√3
Gd-MoS_2	0.06	-0.001	-0.003	-0.007

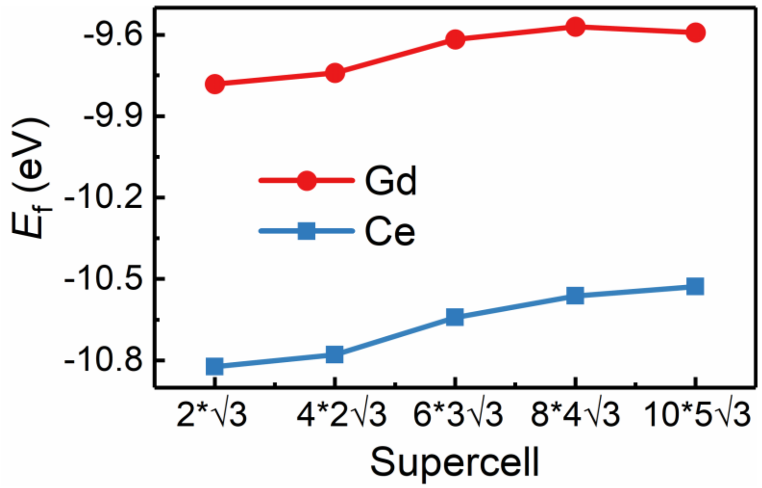

Figure S39. The E_f of Ce- and Gd-MoS_2 with different supercell sizes. Source data are provided as a Source Data file.

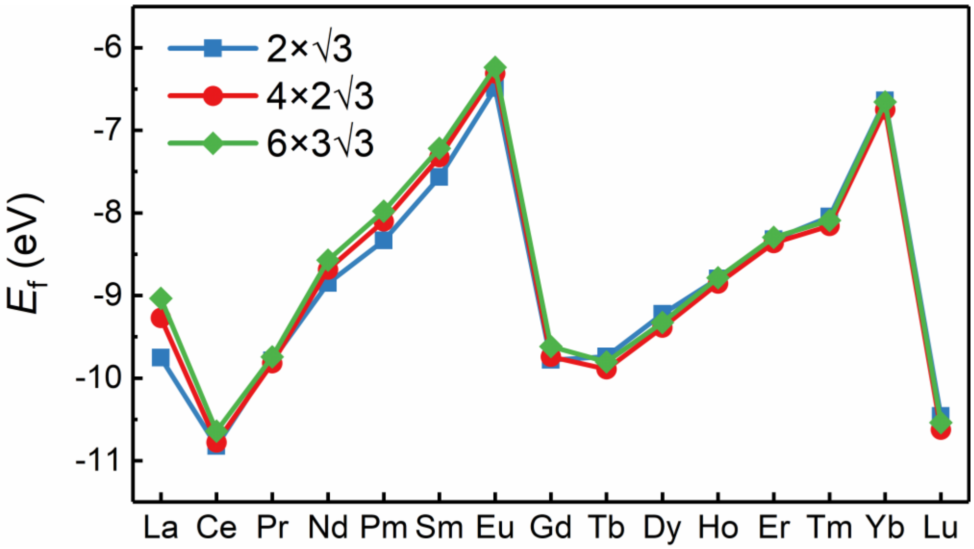

Figure S40. The biperiodic trend of E_f for Ln-MoS_2 with different supercell sizes. Source data are provided as a Source Data file.

(M) Supplementary References

[1] Bai, G.; Yuan, S.; Zhao, Y.; Yang, Z.; Choi, S. Y.; Chai, Y.; Yu, S. F.; Lau, S. P.; Hao, J., 2D Layered Materials of Rare-Earth Er-Doped MoS_2 with NIR-to-NIR Down- and Up-Conversion Photoluminescence. *Adv. Mater.* **2016,** *28*, 7472.

[2] Togo, A.; Tanaka, I., First Principles Phonon Calculations in Materials Science. *Scr. Mater.* **2015,** *108*, 1.

[3] Skelton, J. M.; Burton, L. A.; Jackson, A. J.; Oba, F.; Parker, S. C.; Walsh, A., Lattice Dynamics of the Tin Sulphides SnS_2, SnS and Sn_2S_3: Vibrational Spectra and Thermal Transport. *Phys. Chem. Chem. Phys.* **2017,** *19*, 12452.

[4] Liang, L.; Meunier, V., First-Principles Raman Spectra of MoS_2, WS_2 and Their Heterostructures. *Nanoscale* **2014,** *6*, 5394.

[5] Li, H.; Zhang, Q.; Yap, C. C. R.; Tay, B. K.; Edwin, T. H. T.; Olivier, A.; Baillargeat, D., From Bulk to Monolayer MoS_2: Evolution of Raman Scattering. *Adv. Funct. Mater.* **2012,** *22*, 1385.

[6] Huang, L. F.; Lu, X. Z.; Rondinelli, J. M., Tunable Negative Thermal Expansion in Layered Perovskites from Quasi-Two-Dimensional Vibrations. *Phys. Rev. Lett.* **2016,** *117*, 115901.

[7] Haynes, W. M.; Lide, D. R.; Bruno, T. J., *CRC Handbook of Chemistry and Physics*. 97th ed.; CRC Press: 2016.

[8] Xu, W.; Ji, W.-X.; Qiu, Y.-X.; Schwarz, W. H. E.; Wang, S.-G., On Structure and Bonding of Lanthanoid Trifluorides LnF_3 (Ln = La to Lu). *Phys. Chem. Chem. Phys.* **2013,** *15*, 7839.

[9] Giannozzi, P.; Andreussi, O.; Brumme, T.; Bunau, O.; Buongiorno Nardelli, M.; Calandra, M.; Car, R.; Cavazzoni, C.; Ceresoli, D.; Cococcioni, M.; Colonna, N.; Carnimeo, I.; Dal Corso, A.; de Gironcoli, S.; Delugas, P.; DiStasio, R. A.; Ferretti, A.; Floris, A.; Fratesi, G.; Fugallo, G.; Gebauer, R.; Gerstmann, U.; Giustino, F.; Gorni, T.; Jia, J.; Kawamura, M.; Ko, H. Y.; Kokalj, A.; Kucukbenli, E.; Lazzeri, M.; Marsili, M.; Marzari, N.; Mauri, F.; Nguyen, N. L.; Nguyen, H. V.; Otero-de-la-Roza, A.; Paulatto, L.; Ponce, S.; Rocca, D.; Sabatini, R.; Santra, B.; Schlipf, M.; Seitsonen, A. P.; Smogunov, A.; Timrov, I.; Thonhauser, T.; Umari, P.; Vast, N.; Wu, X.; Baroni, S., Advanced Capabilities for Materials Modelling with Quantum ESPRESSO. *J. Phys. Condens. Matter.* **2017,** *29*, 465901.

[10] Brewer, L., *Systematics and the Properties of the Lanthanides*. First ed.; D. Reidel Publishing Company: 1982.

[11] Huang, L. F.; Grabowski, B.; McEniry, E.; Trinkle, D. R.; Neugebauer, J., Importance of Coordination Number and Bond Length in Titanium Revealed by Electronic Structure Investigations. *Phys. Status Solidi B* **2015,** *252*, 1907.

[12] Nørskov, J. K.; Rossmeisl, J.; Logadottir, A.; Lindqvist, L.; Kitchin, J. R.; Bligaard, T.; Jónsson, H., Origin of the Overpotential for Oxygen Reduction at a Fuel-Cell Cathode. *J. Phys. Chem. B* **2004,** *108*, 17886.

[13] Malcolm W. Chase, J., *NIST-JANAF Thermochemical Tables*. Fourth ed.; American Chemical Society and the American Institute of Physics: 1998.

[14] Henkelman, G.; Uberuaga, B. P.; Jónsson, H., A Climbing Image Nudged Elastic Band Method for Finding Saddle Points and Minimum Energy Paths. *J. Chem. Phys.* **2000,** *113*, 9901.

[15] Hansen, H. A.; Viswanathan, V.; Nørskov, J. K., Unifying Kinetic and Thermodynamic Analysis of $2e^-$ and $4e^-$ Reduction of Oxygen on Metal Surfaces. *J. Phys. Chem. C* **2014,** *118*, 6706.

[16] Xu, D.; Chen, W.; Zeng, M.; Xue, H.; Chen, Y.; Sang, X.; Xiao, Y.; Zhang, T.; Unocic, R. R.; Xiao, K.; Fu, L., Crystal-Field Tuning of Photoluminescence in Two-Dimensional Materials with Embedded Lanthanide Ions. *Angew. Chem.-Int. Edit.* **2018,** *57*, 755.

[17] Tripković, V.; Skúlason, E.; Siahrostami, S.; Nørskov, J. K.; Rossmeisl, J., The Oxygen Reduction Reaction Mechanism on Pt(111) From Density Functional Theory Calculations. *Electrochim. Acta* **2010,** *55*, 7975.

[18] Xing, W.; Yin, M.; Lv, Q.; Hu, Y.; Liu, C.; Zhang, J., *Rotating Electrode Methods and Oxygen Reduction Electrocatalysts*. Elsevier: 2014; Vol. 1, p 1-31.

[19] Greeley, J.; Stephens, I. E.; Bondarenko, A. S.; Johansson, T. P.; Hansen, H. A.; Jaramillo, T. F.; Rossmeisl, J.; Chorkendorff, I.; Nørskov, J. K., Alloys of Platinum and Early Transition Metals as Oxygen Reduction Electrocatalysts. *Nat. Chem.* **2009,** *1*, 552.

[20] Tanaka, H.; Nagahara, Y.; Sugawara, S.; Shinohara, K.; Nakamura, M.; Hoshi, N., The Influence of Pt Oxide Film on the Activity for the Oxygen Reduction Reaction on Pt Single Crystal Electrodes. *Electrocatalysis* **2014,** *5*, 354.

[21] Viswanathan, V.; Hansen, H. A.; Rossmeisl, J.; Nørskov, J. K., Universality in Oxygen Reduction Electrocatalysis on Metal Surfaces. *ACS Catal.* **2012,** *2*, 1654.

[22] Blizanac, B. B.; Ross, P. N.; Marković, N. M., Oxygen Reduction on Silver Low-Index Single-Crystal Surfaces in Alkaline Solution: Rotating Ring Disk$_{Ag(hkl)}$ Studies. *J. Phys. Chem. B* **2006,** *110*, 4735.

[23] Shao, M. H.; Huang, T.; Liu, P.; Zhang, J.; Sasaki, K.; Vukmirovic, M. B.; Adzic, R. R., Palladium Monolayer and Palladium Alloy Electrocatalysts for Oxygen Reduction. *Langmuir* **2006**, 10409.

[24] Schmidt, T. J.; Stamenkovic, V.; Arenz, M.; Markovic, N. M.; Ross, P. N., Oxygen Electrocatalysis in Alkaline Electrolyte: Pt(*hkl*), Au(*hkl*) and the Effect of Pd-Modification. *Electrochim. Acta* **2002,** *47*, 3765.

[25] Stamenkovic, V.; Mun, B. S.; Mayrhofer, K. J.; Ross, P. N.; Markovic, N. M.; Rossmeisl, J.; Greeley, J.; Nørskov, J. K., Changing the Activity of Electrocatalysts for Oxygen Reduction by Tuning the Surface Electronic Structure. *Angew. Chem. Int. Ed. Engl.* **2006,** *45*, 2897.

[26] Stamenkovic, V. R.; Fowler, B.; Mun, B. S.; Wang, G.; Ross, P. N.; Lucas, C. A.; Markovic, N. M., Improved Oxygen Reduction Activity on $Pt_3Ni(111)$ via Increased Surface Site Availability. *Science* **2007,** *315*, 493.

[27] Zhang, J.; Vukmirovic, M. B.; Xu, Y.; Mavrikakis, M.; Adzic, R. R., Controlling the Catalytic Activity of

Platinum-Monolayer Electrocatalysts for Oxygen Reduction with Different Substrates. *Angew. Chem. Int. Ed.* **2005,** *44*, 2132.

[28] Nilekar, A. U.; Mavrikakis, M., Improved Oxygen Reduction Reactivity of Platinum Monolayers on Transition Metal Surfaces. *Surf. Sci.* **2008,** *602*, L89.

[29] Stephens, I. E. L.; Bondarenko, A. S.; Grønbjerg, U.; Rossmeisl, J.; Chorkendorff, I., Understanding the Electrocatalysis of Oxygen Reduction on Platinum and Its Alloys. *Energy Environ. Sci.* **2012,** *5*, 6744.

[30] Huang, L. F.; Scully, J. R.; Rondinelli, J. M., Modeling Corrosion with First-Principles Electrochemical Phase Diagrams. *Ann. Rev. Mater. Res.* **2019,** *49*, 53-77.

[31] Dudarev, S. L.; Botton, G. A.; Savrasov, S. Y.; Humphreys, C. J.; Sutton, A. P., Electron-Energy-Loss Spectra and the Structural Stability of Nickel Oxide: An LSDA+U Study. *Phys. Rev. B* **1998,** *57*, 1505.

[32] Deng, J.; Li, H.; Xiao, J.; Tu, Y.; Deng, D.; Yang, H.; Tian, H.; Li, J.; Ren, P.; Bao, X., Triggering the Electrocatalytic Hydrogen Evolution Activity of the Inert Two-Dimensional MoS_2 Surface via Single-Atom Metal Doping. *Energy Environ. Sci.* **2015,** *8*, 1594.

[33] Li, H.; Tsai, C.; Koh, A. L.; Cai, L. L.; Contryman, A. W.; Fragapane, A. H.; Zhao, J. H.; Han, H. S.; Manoharan, H. C.; Abild-Pedersen, F.; Nørskov, J. K.; Zheng, X. L., Activating and Optimizing MoS_2 Basal Planes for Hydrogen Evolution through the Formation of Strained Sulphur Vacancies. *Nat. Mater.* **2016,** *15*, 48.

[34] Tsai, C.; Li, H.; Park, S.; Park, J.; Han, H. S.; Nørskov, J. K.; Zheng, X.; Abild-Pedersen, F., Electrochemical Heneration of Sulfur Vacancies in the Basal Plane of MoS_2 for Hydrogen Evolution. *Nat. Commun.* **2017,** *8*, 15113.

[35] Yu, L.; Li, F.; Zhao, J.; Chen, Z., Revisiting Catalytic Performance of Supported Metal Dimers for Oxygen Reduction Reaction via Magnetic Coupling from First Principles. *Adv. Powder Mater.* **2022,** *1*, 100031.

[36] Li, Q.-K.; Li, X.-F.; Zhang, G.; Jiang, J., Cooperative Spin Transition of Monodispersed FeN_3 Sites within Graphene Induced by CO Adsorption. *J. Am. Chem. Soc.* **2018,** *140*, 15149-15152.
